# Prediction models for child and adolescent mental health: A systematic review of methodology and reporting in recent research

**DOI:** 10.1002/jcv2.12034

**Published:** 2021-09-24

**Authors:** Morwenna Senior, Thomas Fanshawe, Mina Fazel, Seena Fazel

**Affiliations:** ^1^ Department of Psychiatry Oxford Health NHS Foundation Trust, University of Oxford Oxford UK; ^2^ Nuffield Department of Primary Care Health Sciences University of Oxford Oxford UK

**Keywords:** child protection, justice, multivariable models, risk assessment, risk prediction, self‐harm

## Abstract

**Background:**

There has been a rapid growth in the publication of new prediction models relevant to child and adolescent mental health. However, before their implementation into clinical services, it is necessary to appraise the quality of their methods and reporting. We conducted a systematic review of new prediction models in child and adolescent mental health, and examined their development and validation.

**Method:**

We searched five databases for studies developing or validating multivariable prediction models for individuals aged 18 years old or younger from 1 January 2018 to 18 February 2021. Quality of reporting was assessed using the Transparent Reporting of a multivariable prediction models for Individual Prognosis Or Diagnosis checklist, and quality of methodology using items based on expert guidance and the PROBAST tool.

**Results:**

We identified 100 eligible studies: 41 developing a new prediction model, 48 validating an existing model and 11 that included both development and validation. Most publications (*k* = 75) reported a model discrimination measure, while 26 investigations reported calibration. Of 52 new prediction models, six (12%) were for suicidal outcomes, 18 (35%) for future diagnosis, five (10%) for child maltreatment. Other outcomes included violence, crime, and functional outcomes. Eleven new models (21%) were developed for use in high‐risk populations. Of development studies, around a third were sufficiently statistically powered (*k* = 16%, 31%), while this was lower for validation investigations (*k* = 12, 25%). In terms of performance, the discrimination (as measured by the C‐statistic) for new models ranged from 0.57 for a tool predicting ADHD diagnosis in an external validation sample to 0.99 for a machine learning model predicting foster care permanency.

**Conclusions:**

Although some tools have recently been developed for child and adolescent mental health for prognosis and child maltreatment, none can be currently recommended for clinical practice due to a combination of methodological limitations and poor model performance. New work needs to use ensure sufficient sample sizes, representative samples, and testing of model calibration.

## Graphical abstract

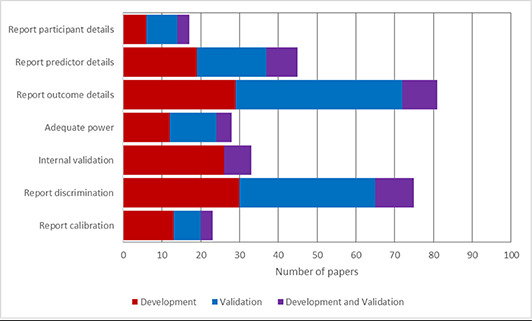

**Key points**

- Many structured risk assessment and risk prediction tools have been developed in the last few years, which may be useful within child and adolescent mental health services. However, few have been translated into clinical practice
- Potential barriers to clinical implementation include poor reporting and methodology
- In this systematic review of recent development and validation studies in child and adolescent mental health, we found that none of the new tools could currently be recommended for use in practice
- Key barriers to clinical utility included inadequate sample sizes in their development, use of samples which are not clinically representative, and poor reporting of model performance (in particular calibration)
- Even when prediction models appeared to perform well, poor methods and reporting meant that it was not possible to conclude that this performance would hold in new populations

## INTRODUCTION

Predicting future outcomes is a core component of practice in child and adolescent mental health, and structured approaches are increasingly sought to inform these judgements. Structured risk assessment tools can contribute to the assessment of suicide risk (Asarnow & Mehlum, 2019), risk of reoffending in juvenile justice settings (Singh et al., 2011; Viljoen et al., 2012), and predicting child maltreatment within child protection services (van der Put et al., 2017). Meanwhile new tools are being developed for different areas of clinical service need, such as identifying children at risk of future mental illness who might benefit from preventive and early interventions (Cohen et al., 2019; Lewis et al., 2019). If implemented, validated models can inform individualised treatment and enable efficient allocation of and access to preventive interventions. For example, the use of structured screening tools for suicide risk has been recommended by the US Joint Commission (healthcare accreditation agency) for all children attending emergency departments in the USA as part of a strategy to reduce suicide rates (DeVylder et al., 2019).

However, there are considerable challenges involved in selecting appropriate tools for use in clinical practice (Larsson, 2021). While new research is utilising complex methodology to develop predictive models (Afzali et al., 2019; Walsh et al., 2018), few have been translated into clinical practice. Instead, many of the currently used tools incorporate only a few risk factors and have not been robustly tested in relevant populations. For example, a recent systematic review of risk assessment tools for self‐harm and suicide found that no single tool was suitable for use in adolescents (Harris et al., 2019). In order to overcome these challenges, there is a need for research which is clinically relevant and methodologically sound, in accordance with recent guidance on best practice for prediction models (Steyerberg & Harrell, 2016; Steyerberg et al., 2013; Wolff et al., 2019). The lack of standardised reporting contributes to difficulties in assessing model performance and generalisability (Collins et al., 2015). Meanwhile methodological problems can lead to ‘overfitting’ the model to the sample in which it was developed, resulting in models which appear to have high predictive accuracy but perform poorly when implemented in new populations (Steyerberg & Harrell, 2016).

In this systematic review, we aimed to assess the clinical utility of recent studies developing or validating multivariable risk models relevant to child and adolescent mental health. Specifically, we examined aspects of methodology and reporting in order to identify barriers to translating new models into clinical practice (Collins et al., 2015; Steyerberg et al., 2013; Wolff et al., 2019). Our aim was to provide an up to date assessment of current research practice after the introduction of latest guidance on methodology (Steyerberg et al., 2013; Wolff et al., 2019), and reporting (Collins et al., 2015; Wolff et al., 2019). We focused on studies since 2018 to identify examples of models in current use and patterns of recent methodology which can be improved on by future research, and which clinicians should be aware of when assessing tools for use in practice. This approach allowed us to capture sufficient studies to reflect the broad scope of recent modelling research and identify common pitfalls in current research practice.

## METHODS

We conducted a systematic review following the Preferred Reporting for Systematic Reviews and Meta‐Analysis guidelines (Moher et al., 2009). We registered the study in a prospective register of systematic reviews (PROSPERO #42020164148).

### Search strategy

We searched five electronic databases: EMBASE, PsycINFO, Medline, Global Health and ERIC. We limited the search to papers published between 1st January 2018 and the search date (18th February 2021) in order to examine current methodological and reporting practices. We used a combination of search terms related to: prognostic models [(“prognostic scor*” or “predict* model*” or “risk assessment” or “risk score” or “risk predict*” or “risk calculator” or “risk model*”) AND (score or scoring or index or model* or predict*)], children and adolescents (child or children or infant or teen* or adolesc* or youth or young or juvenile), mental health and related outcomes, and model development/validation (develop* or derivat* or valid* or predict* or discriminat* or accura* or reliab*). Full search terms are reported in Appendix S1. To identify additional studies, we reviewed references and citing articles for recent systematic reviews on related topics (Harris et al., 2019; van der Put et al., 2017; Viljoen et al., 2012).

### Eligibility assessment

We included studies in all languages, reporting on models developed for all care settings. Inclusion criteria were: (1) model with two or more variables combined in any way, (2) prognostic models: the outcome is not present at the time of prediction, (3) primary purpose of the model is estimating outcome probability for individuals, (4) model designed to be used for children and adolescents aged ≤18 years, or tested in population where >90% are likely to be aged ≤18 years, (5) the outcome is relevant to child and adolescent mental health. We used a broad definition of relevant outcomes in order to incorporate models from multiple disciplines where risk prediction is a routine part of practice. Relevant outcomes included: diagnosis with a mental illness, violence or offending, suicide/self‐harm, substance use and child maltreatment. Studies were excluded if the primary aim was to examine aetiology or individual risk factors. We also excluded models incorporating only neuroimaging or genetic predictors as these are very rarely translated into clinical practice and involve unique methodological challenges outside the scope of this review. However, we included models combining these with other predictor types. Although adolescence can be seen as extending to 24 years of age, we chose an upper age limit of 18 to reflect the population encountered in child and adolescent mental health, juvenile justice, and child protection services which might use the included models. Morwenna Senior screened abstracts and full texts to determine eligibility, any uncertainties were referred to Seena Fazel.

### Data extraction

Morwenna Senior used a standardised form to extract data related to: study and model characteristics (participant details, outcome[s], prediction horizon, data source, study design, model type, number of predictors, model performance); reporting quality using items from the Transparent Reporting of a multivariable prediction models for Individual Prognosis Or Diagnosis (TRIPOD) checklist (Collins et al., 2015); and quality of methodology. To assess the quality of methodology we used items adapted from previous reviews and guidance on good practice for prognostic modelling studies (Bouwmeester et al., 2012; Mallett et al., 2010; Wolff et al., 2019); we extracted information on handling of missing data, selection of predictors, handling of continuous predictors, use of internal validation, number of outcome events and events per candidate predictor, type of model performance measure, and (for validation studies) whether the predictors and risk calculation matched the original model. Morwenna Senior assessed risk of bias using the PROBAST checklist, which assesses risk of bias in four domains (participants, predictors, outcome and analysis) (Wolff et al., 2019). Each domain was assessed as high or low risk of bias. A high risk of bias in any of the four domains led to a judgement of high overall risk of bias.

Studies were separated into three groups: (1) development studies (which report a new multivariable prediction model), (2) validation studies (which test the performance of an existing model in a new population), and (3) studies which include both validation of an existing model and development of a new model. For group 3 studies, data were extracted on the reporting of development and validation components separately, then information was combined to assess reporting in the paper as a whole.

For studies reporting on multiple models, characteristics and performance of the main model were extracted where possible. For TRIPOD items, papers presenting multiple models were required to report the relevant detail for all analyses for it to be recorded as present.

### Data synthesis

To provide an overview of the clinical utility of recent research, model characteristics and performance measures are presented for each model development study, alongside methodological details which are important for assessing the generalisability of a model. Where these were reported, we calculated the events per variable (EPV) based on all candidate variables considered for inclusion in the model (including each category of categorical variables), and the number of events in the dataset used for model development. If the number of candidate variables was not reported, maximum EPV was estimated based on the number of variables in the final model or the number of reported variables. As a rule‐of‐thumb, it has been suggested that there should be a minimum EPV of 10 to ensure adequate sample size for development of a robust model and to prevent overfitting (Peduzzi et al., 1996). We summarised the number of studies meeting this cut‐off. Calculating EPV represents a simplified approach to assessing adequate sample size, which also depends on model type, outcome prevalence, overall model performance and predictor distributions (Riley et al., 2020; Wolff et al., 2019). In addition, the threshold of 10 EPV is subject to ongoing discussion and refinement. For example, prediction models using machine learning techniques may require substantially higher EPVs (often >200) to minimise overfitting (Wolff et al., 2019). Nonetheless, although EPV is to some extent an arbitrary measure, it has been recommended in consensus guidance for assessing risk of bias in prediction model studies and a low EPV can be interpreted as conferring a high risk of bias.

To examine factors which act as barriers to translating multivariable models into clinical practice, we considered aspects of reporting and methodology for both development and validation studies. For each reporting and methodology item, we summarised findings in terms of relative and absolute frequency with which the item was reported, separated by study type.

Posters and oral presentation abstracts were excluded from the quantitative synthesis because they contained insufficient information to assess reporting and methodology.

## RESULTS

We identified 4183 relevant studies from the database search, and three additional studies through other sources. After screening, 125 studies were eligible for inclusion. Of these 125 investigations, 25 poster and oral presentation abstracts were excluded from further analysis due to insufficient information (Figure S1), of which 15 were abstracts reporting new models, and 10 were validation studies. Of 100 remaining studies, 52 studies reported the development of a new model (details and references in Table S1), including six studies developing and externally validating the same model and five studies developing a new model and validating an existing model, or a model developed in cross‐sectional data. Forty eight studies reported on the validation of existing prediction models (full details and references in Table S2).

Most included studies used a cohort design (86 of 100 studies, 86%), six (6%) used a case‐control sample and one used a nested case‐control. Three studies that presented new risk models did not use any data in the model development (these based the models on the expert opinion of the authors or a group of stakeholders) (Kang et al., 2019; Pettit et al., 2018; van Minde et al., 2019). Forty six studies (46%) used prospective data collection, and 49 (49%) utilized retrospective data. Two other studies used a mixed sample of retrospectively and prospectively collected secondary data (Tate et al., 2020; Vincent et al., 2019).

### Novel models for child and adolescent mental health prediction

In model development studies (*k* = 52), the outcome domains most commonly predicted by novel models were mental illness diagnosis (18 studies, 35%), violence or recidivism (*k* = 9, 17%), functional outcomes and symptoms (*k* = 6, 12%), self‐harm or suicide attempts (*k* = 6, 12%) and outcomes relevant to child safeguarding (*k* = 5, 10%). Other models predicted substance use, healthcare service use, and response to medication or psychological treatment. Details of outcomes and participant characteristics for studies reporting the development of new models are presented in Table S1, with details of validation studies in Table S2.

We examined whether new models had undergone validation. Thirty three out of 52 new models (63%) had been tested using internal validation, while six (12%) were published with results from external validation in an independent sample. We identified one tool developed in the 3‐year search period with external validation reported in two separate papers (Rocha et al., 2021). In studies that used internal validation, the commonest methods were random split sample validation (15 studies) and *k*‐fold validation (12 studies).

Measures of model performance including c‐statistic (also known as AUC), calibration and classification measures such as positive predictive value, sensitivity and specificity are presented in Tables S1 and S2. Judgement of the clinical utility of each tool will depend on the proposed application and a balance of performance measures. C‐statistics reported for new models ranged from 0.57 for a tool predicting age‐18 ADHD in an external validation sample to 0.99 for a machine learning model predicting foster care permanency.

Only two studies (one developing a new model and one validating an existing model) were scored as low risk of bias according to the PROBAST checklist (Brathwaite et al., 2020; Caye et al., 2020). In particular, most studies had a high risk of bias in the analysis domain, with only two studies categorised as at low risk of bias in this domain (Tables S1 & S2).

### Reporting of key definitions: Development and validation studies

For all model development and validation studies (*n* = 100), we assessed reporting of eligibility criteria and sample characteristics (see Table 1). Most studies presented participant eligibility criteria and age range. However only 17 studies (17%) reported full sample characteristics (demographics, predictors and outcome prevalence) based on the TRIPOD checklist.

**TABLE 1:** Reporting of key definitions relevant to model generalisability

Key definitions		Development studies	Validation studies	Development and validation
		Number of papers (%) *n* = 41	Number of papers (%) *n* = 48	Number of papers (%) *n* = 11
Participants	Eligibility criteria	38 (93)	46 (96)	10 (91)
	Age range	31 (76)	39 (81)	8 (73)
	Report full sample characteristics	6 (15)	8 (17)	3 (27)
Predictors	Report, define, and describe measurement of all predictors	19 (46)	18 (38)	8 (73)
	List all candidates (development) or all predictors (validation)	27 (67)	26 (54)	8 (73)
	Report method of handling predictors in analysis	24 (59)	n/a	6 (55)
Outcome	Complete reporting of outcome details (definition, measurement, timing of measurement, timing of prediction)	29 (71)	43 (90)	9 (82)
	Report number of outcome events (each analysis)	28 (68)	40 (83)	7 (64)

We examined whether validation and development studies reported all predictors used in analysis and provided relevant details on how these were handled in analyses (see Table 1). Forty five studies (45% of all papers) reported, defined and described the measurement of all predictors. Most studies provided complete details of the outcome being predicted.

### Outcome events

The number of outcome events for each analysis was clearly reported in 75 out of 100 studies. Models developed in samples with small outcome event numbers are at risk of overfitting to the sample. Sixteen of 52 studies (31%) reporting the development of a new model had an EPV ≥10 for the main model (see Table 2). Eighteen (31%) of 59 external validation studies reported ≥100 outcome events in each analysis. Only five papers reported a statistical reason for the sample size used, the remaining papers used all participants in a cohort or did not mention sample size.

**TABLE 2:** Modelling and methodology

		Development studies	Validation studies	Development and validation
Domain		Number of papers (%) *n* = 41	Number of papers (%) *n* = 48	Number of papers (%) *n* = 11
Missing data	Report method of handling missing data	22 (54)	22 (46)	6 (55)
	Report amount of missing data for outcome	17 (41)	13 (27)	3 (27)
Considerations for model overfitting	Report use of internal validation	26 (63)	n/a	7 (64)
	Events per variable (median, IQR, range) for 42 models from 32 papers for development studies*	9.1 (IQR: 1.6–27.0, range: 0.02–455.8)	n/a	3.2 (IQR 2.4–16.3, range 0.3–48.8) (*n* = 9 models from 8 papers)
	EPV>10 (main models) *	12 (29)	n/a	4 (36)
	EPV<10 (main models) *	19 (46)	n/a	4 (36)
	EPV could not be calculated (no report of candidate variables/event number) *	9 (22)	n/a	3 (27)
Modelling methods	Describe all modelling methods/how risk calculated (validation)	12 (29)	37 (77)	3 (27)
	Candidate predictors (*n*) (median, IQR, range)	39 (IQR 17–124, range 2–662)	n/a	16 (range 11‐42)
	Use continuous predictors	31 (76)	n/a	7 (64)
	All continuous predictors handled as continuous (*n* = 31)	10 (24)	n/a	2 (18)
Statistical power: validation studies	Events >100 (smallest event number if multiple analyses)	n/a	12 (25)	6 (55)
Model reporting for replication	Report full model	21 (51)	n/a	6 (55)
	Explain how to use the model	14 (34)	n/a	6 (55)
	Provide simplified score/online calculator/nomogram	10 (24)	n/a	6 (55)

Abbreviation: EPV, events per variable.

* where EPV was not reported, it was estimated based on reported candidate predictors, or variables in the final model if candidate predictors were not reported.

### Modelling methods and data handling

We assessed how missing data was handled and reported in the included studies (see Table 2). Fifty studies (50%) reported the method used to handle missing data. A minority of studies (13%, 13%) used multiple imputation, which is the preferred method unless the amount of missing data is negligible (Collins et al., 2016). The commonest approach was to use complete‐case analysis (31 studies, 31%).

**FIGURE 1:**
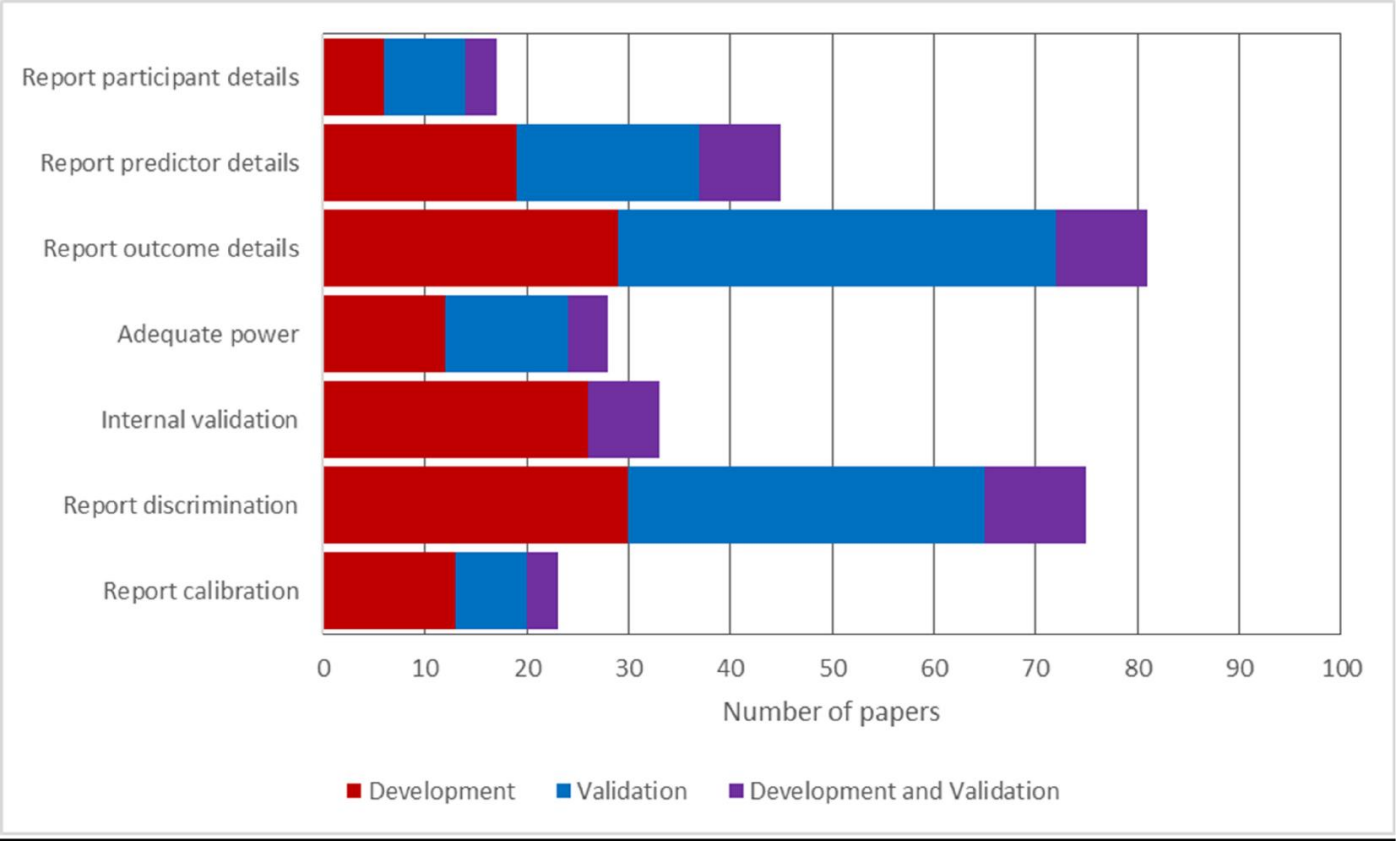
Compliance with reporting and methodology recommendations, by category of paper. Adequate power = events per variable ≥10 for development studies, or total number of events ≥100 for validation studies. Development and validation refers to papers reporting on both model development, and external validation of a new model or existing model

Selection of predictors for inclusion in a multivariable model can lead to the introduction of bias or loss of useful information. Eleven of 52 studies (21%) developing a new model selected predictors based on univariate association with the outcome, which can lead to exclusion of predictors which could contribute to model performance (Collins et al., 2015). We also examined the handling of continuous predictors during modelling, specifically whether continuous predictors were split into categories (Collins et al., 2015; Wolff et al., 2019). We identified 38 development studies (73%) that used continuous predictors, of which 12 (32%) maintained all relevant predictors as continuous in their analyses (see Table 2). Compliance with key recommendations for methodology and reporting for each included development study are summarised in Figure 1 and Table S3.

### Model performance measures

Table 3 shows the type of performance measure reported in included studies. Most papers (*k* = 75, 75%) reported c‐statistic (equivalent to AUC), while only 26 studies (26%) reported some measure of model calibration.

**TABLE 3:** Types of model performance measure reported

	Number of papers reporting measure			
Measure	Total number of studies (*n* = 100)	Development studies (*n* = 41)	Validation studies (*n* = 48)	Development and validation (*n* = 11)
Overall performance measure				
None	85	33	43	9
Brier Score/R2	9	6	2	1
Nagelkerke's R2	4	2	1	1
Discrimination				
AUC/c‐statistic	75	30	35	10
No discrimination measure	22	10	12	0
Calibration				
Plot	12	9	2	1
Hosmer‐Lemeshow	9	5	3	1
Calibration in the large	6	3	2	1
Calibration slope	7	4	2	1
Expected/observed index	4	1	0	3
Other	6	2	3	1
None	77	28	41	8
Classification measure				
Sensitivity	40	18	15	7
Specificity	39	18	15	6
Positive predictive value	30	14	12	4
Negative predictive value	26	10	12	4
Overall accuracy	12	8	2	2
Odds ratio/relative risk	9	2	6	1
Positive likelihood ratio	3	1	2	0
Negative likelihood ratio	4	1	2	1
Comparison measures ‐ incremental value/comparing model performance				
Net reclassification index	2	0	2	0
DeLong AUC comparison	8	0	7	1

*Note*: Overall accuracy = proportion of all binary model predictions which are correct [(True positives + true negatives)/(True positives + false positives + true negatives + false negatives)].

## DISCUSSION

In this systematic review, we provide an overview of methods used in recent research on risk assessment and prediction models for child and adolescent mental health. Specifically, we provide a summary of model development and validation studies from 2018 onwards, with a focus on key aspects of reporting and methodology that will impact on their clinical utility. Several important aspects of reporting and methodology were frequently omitted or poorly implemented. Future research should address these limitations, and clinicians should be aware of the implications when assessing risk prediction tools for use in practice.

### New multivariable models

We identified 52 studies reporting on the development of novel multivariable prognostic models for a variety of outcomes relevant to child and adolescent health. Although we identified some promising tools, none can be recommended for clinical practice due to a lack of robust evidence on predictive performance in clinically relevant populations. Some of the identified tools performed poorly. Even when they appeared to perform well, methodological and reporting limitations made it difficult to conclude that good performance would persist in clinically relevant populations. The most common outcome domain predicted by these models was future diagnosis with a mental illness. Such models could be used in schools or social care settings to identify children who are at risk of mental health problems who might benefit from preventive interventions or surveillance for the emergence of mental illness. Reported model performance varied widely, but should be interpreted in the context of the population in which they were measured: model performance in external validation samples is likely to be poorer than development samples and measures derived without internal validation are unlikely to reflect true model performance.

Examples of promising tools include a logistic regression model predicting the diagnosis and persistence of ADHD in young adulthood using predictors collected before age 12 (Caye et al., 2020). Positive aspects of the study included the use of external validation in three independent samples and an adequate number of events for development and validation. However, the feasibility of its use in practice is unclear as predictions are based on eight items that include IQ and a checklist of 18 ADHD symptoms which are not provided as part of the tool. In addition, the online risk calculator can generate a very high individual percentage risk (up to 90%). At these high risk levels, the estimate will lack precision and outputs should reflect this uncertainty. Poorer model performance in a Brazilian validation sample highlights the importance of external validation when assessing generalisability.

Future research should aim to externally validate existing models and research proposals for new models should incorporate plans for external validation. It will be important to assess how models perform in a variety of contexts, for example exploring whether models developed in high‐income countries can be generalised to populations in low‐ and middle‐income settings. We identified a recent example of research enacting this approach with a model for adolescent depression (Rocha et al., 2021). The authors developed a multivariable model for adolescent depression using a Brazilian population cohort. The model was then updated and validated in two independent samples from the UK and New Zealand, and subsequent papers report the results of external validation studies examining the performance of this model in Nepalese and Nigerian contexts (Brathwaite et al., 2020, 2021).

Several new multivariable models were developed in high‐risk samples, which may represent a more clinically feasible approach than population screening and could help to identify specific modifiable risk factors within selected populations. Examples of this approach include models developed in a sample of children who have experienced trauma or victimisation, predicting psychosocial and economic outcomes (Latham et al., 2019), psychiatric disorders (Meehan et al., 2020), and PTSD (Lewis et al., 2019), and models predicting suicide attempts within the following 90 days for individuals with a diagnosis of a mental health problem (Simon et al., 2018).

### Barriers to clinical utility

Many of the studies we identified were underpowered, presenting an important barrier to the development of robust, clinically useful tools. Required sample size for development of a robust prognostic model depends on the number of outcome events and candidate predictors, but also the total number of participants and expected model performance (Riley et al., 2020). As a rule of thumb, a minimum of 10 EPV has been suggested for adequate power (Peduzzi et al., 1996), but we found that only around a third of model development studies met this threshold. In addition, many model development studies used random split‐sample techniques for internal validation, which is not recommended as it results in less data for model development (Austin & Steyerberg, 2017). Models developed in underpowered samples are at risk of overfitting—they may have good apparent performance in development samples but perform poorly in different populations, compromising accuracy when tools are applied to heterogeneous clinical practice. Statistical power and overfitting are especially important when evaluating predictive models based on ‘data‐driven’ techniques which use a large number of candidate predictors (Van Der Ploeg et al., 2014). An example of this was a model that used machine learning techniques to predict suicide attempts using data from routine health records that included over 600 candidate predictors (Walsh et al., 2018). Although this study reported excellent model performance in terms of discrimination (AUC 0.96), this should be interpreted very cautiously without validation in an external sample. External validation studies were also frequently underpowered, with just 28% having more than the recommended minimum of 100 outcome events in each analysis (Collins et al., 2016).

Another important consideration when assessing how models might translate into clinical practice is whether the sample used is representative of real‐world populations. A minority of studies reported full characteristics of the sample used. This makes it difficult for clinicians to evaluate applicability to their field of practice. Some studies used selected samples which might produce biased estimates of model performance. For example, one investigation utilised a case control study design to assess model performance for predicting suicide attempts compared to a general population control sample which excluded individuals with self‐harm that was not life‐threatening (Walsh et al., 2018). Another study which reported very good model discrimination used a cohort of children who exited foster care (Elgin, 2018). The model predicted whether the child entered a permanent placement (including reunification with original caregiver or adoption) on exiting care. Such a tool might be most useful for children entering or currently in foster care, but these populations are likely to differ from the exit cohort used for model development. In the context of this limitation and a lack of detail on other aspects of study design (such as the timing of predictor measurement), the near‐perfect AUC of 0.99 reported should therefore also be interpreted with considerable caution.

Many studies do not adequately report the range of performance measures that is necessary to make informed decisions about clinical utility. The relative importance of different measures will depend on the specific context within which the model might be used (Steyerberg et al., 2010). As a minimum, both discrimination (whether a patient who has the outcome has a higher risk prediction than one who does not) and calibration (how close expected outcomes based on model predictions are to observed outcomes) should be reported (Collins et al., 2015; Steyerberg et al., 2010; Wolff et al., 2019). Calibration is particularly important for tools where predictions are presented as a probability of the outcome, which was the case for 26 of the included studies. However, we found that most model development and validation studies did not report any measures of model calibration.

Transparency in reporting may also have implications for the acceptability of prediction models. For example, the fairness of prediction models used within child protection services and juvenile justice systems has come under scrutiny (Hao & Stray, 2019; Keddell, 2019; Pegg & McIntyre, 2018), with concern that they might reinforce bias and inequality. We identified two studies in Canadian offenders which aimed to address such concerns by testing the predictive performance of models used in the criminal justice system in subpopulations of offenders defined by ethnicity (Li et al., 2020; Muir et al., 2020). Concerns about fairness have been exacerbated by a lack of transparency because details of modelling methodology, which are sometimes proprietary, are not always available for scrutiny. Considerations of fairness and acceptability could be particularly important if predictive models are used to determine the allocation of preventive resources and are pertinent for child and adolescent mental health in light of important influences that social and familial factors play in mental health.

### Strengths and limitations

Although previous systematic reviews have summarised multivariable prediction tools available for specific clinical presentations or domains (Harris et al., 2019; van der Put et al., 2017; Viljoen et al., 2012), to our knowledge, this is the first review to assess key aspects of reporting and methodology within the field. To do this, we applied established, expert guidance on good practice. This approach can enable clinicians to make informed assessments regarding the utility of new models and highlight key considerations that can improve the quality of future research. One strength of our approach was the broad inclusion criteria for relevant outcomes which enabled us to include studies from child protection and juvenile justice. This allowed us to incorporate insights from different research domains and reflect the multidisciplinary nature of child and adolescent mental health. On the other hand, in the context of this broad search it is possible that some relevant studies were not identified.

A limitation of our search strategy was that we focused on recent publications in order to provide an overview of current research practice. As a result, our review should not be seen as a comprehensive overview of models which are available for use in practice, but instead complements and updates previous reviews that provided a synthesis on evidence for existing tools (Harris et al., 2019; van der Put et al., 2017; Viljoen et al., 2012). One implication of our focus on recent research is that external validation studies may have been carried out or planned for novel tools but not yet published. As such, although at present we could not recommend any of the included models for uptake in clinical practice, future validation studies may provide implementation support for some tools. A further limitation of our study was that a single author (Morwenna Senior) completed screening, study selection and data extraction. Potential bias was minimised by referring all uncertainties to the senior author (Seena Fazel), acting independently, and using standardised data extraction templates.

It is also important to note that we have focused on reporting and quality of methodology, but other considerations are also important for assessing the clinical utility of prediction models. Tools should provide clinically useful information, be affordable and accessible, have clearly defined items which can be easily collected and reliably completed and the feasibility of their use in clinical practice should be evaluated (Fazel & Wolf, 2018; Oliver et al., 2021). In addition, it will be important to assess the acceptability of tools for children, adolescents and their caregivers. Acceptability may in turn be influenced by perceived or actual stigma associated with being deemed high risk of future mental illness. One further limitation is that although our search used Global Health, a database with international coverage, we identified few studies from lower‐ and middle‐income countries and it is possible that some studies from these settings were missed.

### Conclusion

Recent research has produced several promising prediction models but current evidence does not support their translation into clinical practice. A common focus of these tools, which may have a significant impact on clinical practice, is the prediction of future mental illness. Another common approach is the development of tools for high‐risk populations which may represent a more feasible option than population screening. With a stronger evidence base, these tools could be useful for planning and targeting preventive and early interventions. Inadequate sample size, lack of validation in representative samples, and partial reporting of model performance measures may be limiting possibilities to translate research into clinical practice. Researchers need to ensure that these key issues are addressed. Meanwhile, funding bodies may have a role in ensuring that basic criteria (such as adequate sample size, appropriate validation and an analysis plan that includes assessment of discrimination and calibration) are met in order to incentivise clinically relevant research.

## CONFLICTS OF INTEREST

Seena Fazel is a member of the Editorial Advisory Board for JCPP *Advances*. The remaining authors have declared that they have no competing or potential conflicts of interest. [Corrections made on 22 June 2022, after first online publication: This Conflicts of Interest statement has been updated in this version.]

## ETHICS STATEMENT

Formal ethical approval was not required as this study used only secondary data from previously published studies.

## AUTHOR CONTRIBUTION

Morwenna Senior and Seena Fazel conceived the study; Morwenna Senior, Thomas Fanshawe, Mina Fazel and Seena Fazel contributed to study design. Morwenna Senior carried out searches, data collection and data analysis with supervision from Seena Fazel. Thomas Fanshawe, Mina Fazel and Seena Fazel contributed to interpretation of findings. Morwenna Senior drafted the paper and all authors critically reviewed it. Seena Fazel provided overall supervision. We are grateful to Dr Rongqin Yu for help carrying out searches.

## Supporting information

Supporting Information S1

## Data Availability

The study used secondary data extracted from published studies. Data used in the study is available on request from Morwenna Senior.
