# Supplementary material for: Prediction models for child and adolescent mental health: A systematic review of methodology and reporting in recent research
Source: JCPP Adv. 2021 Sep 24;1(3):e12034. doi: 10.1002/jcv2.12034 (PMC10242964; doi:10.1002/jcv2.12034)
Supplement: Supplementary file 1 — Supporting Information S1 [file JCV2-1-e12034-s001.docx]

# Appendix: Multivariable prediction models for child and adolescent mental health: a systematic review of methodology and reporting.

Contents

##

[Appendix: Multivariable prediction models for child and adolescent mental health: a systematic review of methodology and reporting. 1](#_Toc80871134)

Appendix S1: [Search string for Embase/psycINFO/Medline/Global Health 2](#_Toc80871135)

[Figure S1: Flow diagram of systematic search for studies 3](#_Toc80871136)

[Table S1: Overview of recent models relevant to child and adolescent mental health 4](#_Toc80871141)

[Table S2: Details of included validation studies 18](#_Toc80871142)

[Table S3: compliance with reporting and methodology recommendations for included model development studies 24](#_Toc80871143)

[Note: ✓ indicates that full details are reported (as per TRIPOD guidance) or that key methodological recommendations were reported as met. * = tool developed without data (eg. based on expert opinion) 26](#_Toc80871144)

[References 27](#_Toc80871145)

## Appendix S1. Search string for Embase/psycINFO/Medline/Global Health

All searches were performed as keyword searches (includes title, abstract, subject headings).

| Search Number | Search term(s) |
| --- | --- |
| 1 | "prognostic scor*" |
| 2 | "predict* model*" |
| 3 | "risk assessment" |
| 4 | "risk score” |
| 5 | "risk predict*" |
| 6 | "risk calculator" |
| 7 | "risk model*" |
| 8 | (score or scoring or index or model* or predict*) |
| 9 | 1 or 2 or 3 or 4 or 5 or 6 or 7 |
| 10 | 8 and 9 |
| 11 | Child |
| 12 | Children |
| 13 | Infant |
| 14 | Teen* |
| 15 | Adolesc* |
| 16 | Youth |
| 17 | Young |
| 18 | Juvenile |
| 19 | 11 or 12 or 13 or 14 or 15 or 16 or 17 or 18 |
| 20 | (anxiety or "obsessive compulsive" or OCD or phobia or "phobic disorder*" or "panic disorder*") |
| 21 | ("stress disorder*" or PTSD) |
| 22 | ("eating disorder*" or "anorexia nervosa" or "bulimia nervosa" or pica) |
| 23 | (depression or depressive or "bipolar disorder*" or "mood disorder*" or "affective disorder*") |
| 24 | ("mental health" or "mental illness") |
| 25 | (psychopathology or psychiatr*) |
| 26 | "somatoform disorder*" |
| 27 | ((attention adj4 disorder*) or ADHD) |
| 28 | ((behavio* adj4 disorder*) or "conduct disorder*") |
| 29 | "personality disorder*" |
| 30 | (autis* or "asperger syndrome*" or ASD) |
| 31 | (schizophren* or psychot* or psychos*) |
| 32 | ("self-harm*" or "self harm*" or "self-inj*" or NSSI or overdos* or suicid*) |
| 33 | (crime or offend* or offenc* or delinquen* or violen* or "antisocial behav*" or recidiv*) |
| 34 | (abuse or maltreat* or welfare or CPS or "child protection" or neglect) |
| 35 | 20 or 21 or 22 or 23 or 24 or 25 or 26 or 27 or 28 or 29 or 30 or 31 or 32 or 33 or 34 |
| 36 | (develop* or derivat* or valid* or predict* or discriminat* or accura* or reliab*) |
| 37 | 10 and 19 and 35 and 36 |
| 38 | Limit 37 to yr="2018 -2019" |
| 39 | Remove duplicates from 38 |

## Figure S1: Flow diagram of systematic search for studies

Abstract-only studies: 25 (15 development, 10 validation)

Studies included in qualitative synthesis: 100

Development: 67, of which 11 include development + validation

Validation: 58

Full-text articles excluded, with reasons
(n =326)

Adult participants: 117

Not prospective: 74

Aim to examine individual risk factors or theoretical model: 57

Variables not combined or only genetic/neuroimaging: 20

Outcome not relevant: 18

Poster/presentation abstract, unable to determine inclusion: 19

Review/commentary/protocol: 11

Data duplicated elsewhere: 7

Unable to access (dissertation): 2

Aim is service description: 1

Records after duplicates removed

n = 3288

Records excluded after title/abstract screening
n = 2837

Full-text articles assessed for eligibility
n = 451

Additional records identified through other sources
n=3

Records identified through database searching
n = 4183

## Identification

## Eligibility

## Included

## Screening

## Table S1: Overview of recent models relevant to child and adolescent mental health

| Study ID and tool name | Outcome and timing | Population | Model type (output) | Validation | Items in final model | EPV | Simplified format/ online calculator | AUC / c-statistic | Calibration | Classification | Risk of bias  **Overall (domains at high ROB)** |
| --- | --- | --- | --- | --- | --- | --- | --- | --- | --- | --- | --- |
| JUSTICE |  |  |  |  |  |  |  |  |  |  |  |
| Barnes2018  **The protective factors for reducing juvenile reoffending measure (PFRJR)** | General reoffending over 7 months | Justice-involved youth, mean age 15 | Additive rating scale  (total score) | None | 15 | 0.6 | No | 0.64 (0.56 - 0.72) | Not reported | Not reported | High (1/4: A) |
| Choi 2020  **Models for 1) any reoffending, and 2) sexual reoffending** | 1. General reoffending, and 2. sexual reoffending within 24 months | Male juvenile offenders in rehab program | Boosted logistic regression (total score, risk groups) | Internal | 1) 47  2) 40 | 1) 13.6  2) 0.34 | Yes | 1) 0.67  2) 0.70 | Not reported | Not reported | High (1/4: A) |
| Kang2019  **Treatment needs and progress scale (TNPS)** | "behavioural and emotional adjustment" not defined | Juvenile sexual offenders, age 12-24y | Structured professional judgement (output type not reported) | None | 45 | Developed without data | No | Not reported | Not reported | Not reported | High (4/4) |
| McKay 2019  **Montana Juvenile Probation Risk Screener (MJPRS)** | Reoffending (any new citation) within one year | Youth involved with juvenile justice system in Montana, mean age 14 | Additive, weighted and unweighted tested (Total score and risk categories) | Internal | 7 | 4.1 | Yes | 0.73 (non-weighted), 0.74 (weighted) | Not reported | Not reported | High (3/4: Pa, O, A) |
| Norris2018  **Caredigion Youth Screening Tool (CYSTEM)** | Offending over 6 months | Referred to justice and prevention service, age 11-18y | Additive  Rating scale; (binary) | None | 6 | Developed in cross-sectional data | Yes | 0.64 (0.55 - 0.74) | Not reported | Sens: 0.89, Spec: 0.34 | Dev: High (3/4: Pa,O,A)  Val: High (2/4: O,A) |
| Ozkan2019 | Sexual re-offending over 2 years | Juvenile Sexual offenders, age not reported, | Machine learning – random forest (probability) | Internal | 336 | 0.6* | No | 0.71 (0.65 - 0.78) | Not reported | Not reported | High (3/4: Pa,Pr,A) |
| Ting2018 | Reoffending, follow-up 2.2-7.3y, median 4.82 | Youth offenders, age 12-19y | Machine learning – random forest (probability) | Internal | 129 | Not reported | No | 0.69** | Not reported | Not reported | High (2/4: Pa,A) |
| Van der Put 2020  **Actuarial risk and needs assessment instrument for truancy (ARNIT)** | Conviction for truancy offence within 2 year | Juvenile offenders assessed with NIJ risk assessment tool by probation services, Netherlands, age 12-18y | Decision tree (Risk groups, % risk) | Internal | 23 | 2.25 | Yes | 0.69 (0.66–0.72) | Not reported | Not reported | High (2/4: O, A) |
| Villanueva2019  **YLS/CMI brief version** | General reoffending over 2 years | Juvenile offenders, age 14-19y | Regression – logistic (probability) | None | 7 | 2.4 | No | 0.79** | Not reported | Sens:0.70, Spec: 0.79 | High (1/4: A) |
| FUTURE DIAGNOSIS | |  |  |  |  |  |  |  |  |  |  |
| Birmaher2018  **COBY risk calculator** | BPD diagnosis within 5 years | Youth at high risk of BPD (diagnosis of BPD NOS +/- family history); age 6-17y | Regression - baseline resetting cox proportional hazards (probability) | Internal and External | 8fi3 | 3.2* | Online calculator | 0.75** | Hosmer-Lemeshow: p=0.56  Calibration plot | Range of values presented | High (2/4: O,A) |
| Cohen2019 | Depression diagnosis over 2 years | Community sample, no previous depression, age 7-27y, performance for age10-15y | Additive rating scale  (total score) | Internal and External | 3 (based on ~58 items from scales) | 2.82 | No | 0.68 | E/O index: 1.18.  Calibration plot | Sens: 0.59, Spec: 0.69, PPV: 0.15, NPV: 0.95 | High (1/4: A) |
| Hamasaki 2021  **Child psychosis-risk screening system (CPSS)** | Schizophrenia diagnosis age 20-29 | Adults with schizophrenia , controls with no psychiatric disorder. predictors retrospectively assessed for age 6-8 | Logistic regression (probability) | None | 8 | 6.75 | Online calculator | 0.83 (0.76-0.89) | Hosmer-Lemeshow 2.82, p 0.90 | Overall accuracy 0.86 | High (3/4: Pr, O, A) |
| Koning2019 | First diagnosis with mental health problem over 12 months | Community sample, 3x models for ages: 1-3y, 4-11y, 12-19y | Regression – logistic (probability) | Internal | 1-3y: 14  4-11y: 14  12-19y: 13 | 1-3y: 117.4  4-11y: 455.8  12-19y: 330.4 | No | 1-3y: 0.63  4-11y: 0.62  12-19y: 0.63 | Calibration plot | Not reported | High (4/4) |
| Lewis2019  **PTSD risk calculator** | PTSD diagnosis by age 18 | Trauma-exposed, age 5-12y | Regression - logistic (probability) | Internal | 12 | 13.3 | No | 0.74** | Cal-in-the-large: 0.1 Calibration slope: 0.9  Calibration plot | Not reported | High  (1/4: A) |
| Meehan2020 | Past-year psychiatric disorder at age 18. | Victimised children, age 5-12y | Regression - LASSO logistic (probability) | Internal | 17 | 15.2 | No | 0.69 (0.64 - 0.73) | Cal-in-the-large: 0.02 Calibration slope: 0.96  Calibration plot | Sens: 0.63, Spec 0.60,  PPV 0.71,  NPV 0.51 | High (2/4: O,A) |
| Moore 2021  **Penn Psychosis Risk Calculator** | Diagnosis with psychosis spectrum disorder, unclear timing | Children receiving paediatric care at clinical high risk for psychosis, and non-high-risk controls. Age 8-21y | Ridge regression (log odds/probability) | None | 6 (includes multi-C-GAS) | Not reported | Yes | 0.68 (apparent performance) | Not reported | Sens: 0.730 (0.713 - 0.748). Spec: 0.663 (0.641 - 0.685) | High (1/4: A) |
| Nichols2018 | First depression diagnosis over 12 months. | Community sample, no previous psychiatric disorder, age 15-18y | Regression – logistic (probability) | Internal | 18 | Male: 79.2  Female: 200.9 | No | Male: 0.71 (0.70 - 0.73)  Female: 0.72 (0.71 - 0.73) | Not reported | Not reported | High (4/4) |
| Pijl2019 | Diagnosis of ASD at 36 months old | Siblings of children with ASD, age 8-36 months | Machine learning - Support vector machines (binary) | None | 28 | 0.9 | No | 0.72 (0.57 - 0.84) | Not reported | Sens: 0.85, Spec: 0.58, PPV 0.30,  NPV: 0.95 | High (2/4: O,A) |
| Rabelo-da-Ponte 2020 | Bipolar disorder diagnosis at age 22 | Birth cohort Pelotas, Brazil. models for perinatal, 11y, 15y 18y old. | Elastic net machine learning model (probability) | Internal | 7 (age 15) to 26 (age 18) | Perinatal – 23.2  11y: 28.3  18y: 9.8 | No | Perinatal 0.62 (0.55–0.69)  11yo 0.64 (0.55–0.72)  18yo 0.82 (0.75-0.88) | Not reported | 18yo model sens 0.72, spec 0.77, PPV 0.18, and NPV 0.97 | High (1/4: A) |
| Rahman 2020 | Diagnosis of ASD, timing unclear | Population-based cohort | Logistic regression (LR), artificial neural network (NN), and random forest (RF) (high/low risk groups) | Internal | 178 | 7.85 | No | LR: 0.726,  RF: 0.693,  NN: 0.709 | Not reported | LR: Sens 0.35, spec 0.97, PPV 0.34  RF: sens 0.24, spec 0.99, PPV 0.57.  NN: sens 0.30, spec 0.98, PPV: 0.572 | High (3/4: Pr, O, A) |
| Rocha 2021 | Depression diagnosis in late adolescence | Development: birth cohort Pelotas, Brazil. Validation: twin birth cohort UK, and birth cohort New Zealand  Age 12-15y | Penalized logistic regression (probability) | Internal and External | 12 | 1.68 | No | Int: 0.71.  Ext UK: 0.62, Ext NZ: 0.67 | Cal slope Int: 1.00, UK: 1.20, NZ: 1.24 | Not reported | High (1/4: A) |
| Sacrey2018  **Autism Parent Screen for Infants (APSI)** | Diagnosis of ASD at 36 months old | Siblings of children with ASD, age 6-24 months | Additive rating scale (total score, binary) | None | 26 | 0.8 | Checklist | 0.78 (0.62 - 0.94) | Not reported | Sens: 0.67, Spec 0.86,  PPV 0.47,  NPV: 0.83 | High (2/4: O,A) |
| Tate 2020 | Mental health problems at age 15 | Population-based twin sample, Sweden, predictors assessed 0 – 14 years | Logistic regression (LR), random forest (RF), support vector machine (SVM), neural network (NN), XG Boost  (binary prediction) | Internal | 85 | Not reported | No | LR: 0.70 (0.67–0–73),  RF: 0.74 (0.71–0.77),  SVM: 0.74 (0.71–0.77) | Not reported | Eg. random forest: NPV 96%, PPV 15% | High (1/4: A) |
| Caye2020  **Prodah risk tool** | ADHD diagnosis at age 17/18 | Community cohorts/treatment trial, age range not reported | Regression - logistic (probability) | Internal and External | 9 | 30.4 | Online calculator | 0.57 - 0.76** | Calibration plot | Range of values presented | Dev: Low  Val: High (2/4: Pr,O) |
| Malda2019 | Conversion to psychosis over 2 years | Adolescents at clinical high risk of psychosis, age 14-40 (development), 14-17 (validation) | Regression -parametric survival model (probability) | Internal external cross-validation | Not reported | 48.2 | No | Age 14-17y: 0.74 (0.28 - 0.95) overall = 0.655 (0.627 - 0.682) | Calibration slope: 0.66  Calibration plot | Not reported | High (2/4: O,A) |
| Hankin2018 | Depressive episode over 18 months | Community sample, age 10-16y | Risk matrix (risk groups) | None | Not reported | 20.7 | No | Not reported | Not reported | Not reported | High (2/4: O,A) |
| Van Meter 2021 | Diagnosis with bipolar spectrum disorder, timing unclear | Development sample: treatment seeking youth with symptoms of mania age 6-12y and matched controls,  Validation: Offspring of parents with bipolar disorder | Cox regresion (probability) | External and Internal | 2 (multi-item scales) | 16.3 | No | 0.73 (ext. validation) | Spiegelhalters z=-6.02, p<0.0001 | External validation: sens 0.76, spec: 0.52, PPV 0.1 | High (1/4: A) |
| CHILD PROTECTION | |  |  |  |  |  |  |  |  |  |  |
| Elgin2018 | Foster care permanency, timeframe not reported | Children exiting foster care, age 0-20y | Machine learning - boosted tree (binary) | Internal | 189 | 105.9 | No | 0.99** | Not reported | Sens: 0.88, Spec: 0.99 | High (3/4: Pa,Pr,A) |
| Hansmann 2020  RIC: risk indication in child sexual abuse and RIC:SV (screening version) | Sexual offence recidivism within 5y – intended for assessment of child sexual abuse cases by CPS | Adults convicted of contact child sexual offences in Austria (intended for use by CPS) | Additive rating scale | None | 9 (6 in screening version) | 1.88 | No | RIC total score - 0.84 (0.76-0.91). RIC-SV: 0.78 (0.69-0.86) | Hosmer-Lemeshow p=0.79 | Not reported | High (3/4: Pa, Pr, A) |
| Thurston2018 | Severe maltreatment causing death or hospitalisation, timeframe not reported | Previous referral to child welfare, age <6y | Decision tree (odds ratio, binary) | None | 4 | 23.3 | No | Not reported | Not reported | Sens: 0.71, Spec: 0.49 | High (4/4) |
| Walsh 2020 | >2 adverse childhood experiences by age 5 months | Birth cohort from New Zealand | Logistic regression (and high/low risk groups) | Internal | 28 | Not reported | No | 0.76 | Not reported | Not reported | High (2/4: O, A) |
| Vaithianathan2018  **Maltreatment risk score** | Model development: Substantiated maltreatment by age 5 Model performance tested for infant mortality | General population, neonates | Regression - logistic (risk groups) | None | 14 | Not reported | No | Maltreatment outcome: 0.88 (0.87 - 0.89) | Not reported | Not reported | High (4/4) |
| SELF-HARM |  |  |  |  |  |  |  |  |  |  |  |
| Hill2019 | Suicide attempt over 12 months | School cohort, age 11-21y | Decision tree (risk groups) | None | 7 | 0.6 | No | Not reported | Not reported | Sens: 0.70, Spec: 0.86  (range reported) | High (2/4: O,A) |
| King2019 | Suicide attempt/death over 3 months | Attending ED, age 12-17y | Regression -– logistic (probability) | Internal | 4 | 1.3 | No | 0.87** | Not reported | Not reported | High (2/4: O,A) |
| Pettit2018 | Suicide, timeframe not specified | Not reported | Decision tree (risk groups) | N/a | 4 | Developed without data | Algorithm | Not reported | Not reported | Not reported | High (4/4) |
| Simon2018 | Suicide attempt or death by suicide during 90 days after healthcare contact | Mental health diagnosis,  1: primary care  2: secondary care  age 13-65 (model performance for age 13-19) | Regression - LASSO logistic (probability) | Internal | 1: 102  2: 94 | 1: 109.2  2: 278.3 | No | 1: 0.83**  2: 0.83** | Calibration plot | Not reported | High (4/4) |
| Su 2020 | Suicide attempt, multiple timepoints from 0-365 days | Children attending for hospital inpatient/outpatient or emergency care. Age 10-18y. | logistic regression LASSO penalization | Internal | 90-day prediction window: 15,  365 days: 13 | Not reported | No | 365 days: 0.81 (0.78–0.85) | Not reported | 365d: sens 0.38 at spec 0.95, PPV 0.05 | High (3/4: Pr, O, A) |
| Walsh2018 | Suicide attempt over 90 days | General population, no self-harm/depression. Mean age 15 | Machine learning - random forest (risk groups) | Internal | Not reported | 1.5 | No | 0.96 (0.95 - 0.97) | Calibration plot | Spec: 1.0, PPV: 0.87 | High (4/4) |
| SYMPTOMS AND FUNCTIONAL OUTCOMES | | | | |  |  |  |  |  |  |  |
| Birmaher 2020 | Recurrence of depressive or hypo/manic episode within 5 years | Youth with bipolar affective disorder, with 2 months of no/minimal mood symptoms after recent episode. Mean age 12 | Boosted multinomial classification trees. | Internal | 12 | Not reported | Online calculator | 0.82 (0.81 - 0.84) | Hosmer-Lemeshow 5.33 | Optimal sens: 0.74, PPV 0.78 (at predictive threshold of 0.5) | High (2/4: O, A) |
| Latham2019 | Psychosocial disadvantage/ economic disadvantage at age 18 | Victimised children, age 5-12y | Regression - LASSO logistic (probability) | Internal | 12 | Psych: 10.7  Econ: 14.3 | No | Psych: 0.65 (0.6 - 0.7)  Econ: 0.8 (0.76 - 0.84) | Psych: cal-in-the-large: 0.07, Calibration slope 0.84,  Econ: cal-in-the-large 0.01, slope: 1.11 | Psych:  Sens: 0.61, Spec: 0.64  PPV: 0.5  Econ:  Sens:0.69  Spec: 0.79  PPV 0.74 | High (2/4: O,A) |
| Li2019 | Behavioural problems after 3 years | Children having surgery for pectus excavatum, age 10 | Regression -– logistic (probability) | None | 4 | 2.7 | Yes | 0.88** | Hosmer-Lemeshow: p= 0.252  Calibration plot | Sens: 0.87, Spec: 0.84 | High (3/4: Pa,O,A) |
| Shui2018 | Sleep problems at 1 year | Children with ASD or pervasive developmental disorder - NOS, age 2-17y, USA and Canada | Regression – logistic (probability) | Internal | 5 | 1.1 | No | Not reported | Not reported | Sens: 0.80 (0.69–0.89), Spec: 0.33 (0.29–0.38), PPV: 0.19 (0.14–0.23), NPV: 0.90 (0.84–0.94) | High (3/4: Pa,O,A) |
| Goodwin2019 | Aggression in next 60 seconds | Inpatients with ASD diagnosis, age 6-17y | Regression - ridge regularized logistic regression (binary) | Internal | unclear | No | No | 0.71** | Not reported | Not reported | High (1/4: A) |
| VanMinde2019  **Postnatal Rotterdam Reproduction risk reduction checklist (R4U)** | Poor development inc. ASD/ADHD | All neonates, age 0-8weeks, Netherlands | Additive - weighted sum (risk groups) | None | 41 | Developed without data | Checklist | Not reported | Not reported | Not reported | High (4/4) |
| SUBSTANCE USE |  |  |  |  |  |  |  |  |  |  |  |
| Afzali2018 | Alcohol ³monthly after 3-4 years | School cohort, mean age 13 | Regression - elastic net (output type not reported) | Internal and External | Not reported | Yes | No | 0.70** | Not reported | PPV: 0.50, NPV: 0.87 | Dev: High (1/4: A)  Val: high (2/4: O,A) |
| Sylvestre2018 | Smoking initiation within 12 months | Community sample, never-smokers, age 12-16y | Regression - LASSO logistic (probability) | Internal | 12 | 6.4* | Yes – online calculator and simplified scoring sheet | 0.77** | Calibration plot | Sens: 0.8, Spec: 0.55 | High (1/4: A) |
| Zhang-James 2020 | Substance use disorder age 18-19 (various prediction horizons used) | Population-based cohort with no substance use disorder, age 17y | Random forest (main), also test Neural Network (LSTM) | Internal | 10-266 | Main: 4.2, smallest 0.02 | No | Random forest: 0.67 (0.64-0.71) | Hosmer–Lemeshow test p value 0.13 | Sens 0.27, and PPV 0.20. | High (3/4: Pr, O, A) |
| OTHER |  |  |  |  |  |  |  |  |  |  |  |
| Grebla2019  **Suboptimal Response Prediction Model** | Suboptimal response to ADHD medication (MPH) over 12 months | ADHD diagnosis, recently initiated methylphenidate or lisdexamfetamine, age 6-17y | Regression - group LASSO (probability, risk groups) | Internal | Not reported | Not reported | No | Not reported | Not reported | Not reported | High (4/4) |
| Ogrim2019  **ADHD Global Scale** | Response to ADHD medication at 4 weeks | ADHD diagnosis, IQ >70, age 8-17y | Additive - weighted sum (total score) | None | 6 | 1.5* | No | 0.91 (0.87 - 0.95) | Not reported | Sens: 0.86, Spec: 0.88 | High (1/4: A) |
| Evers2018 | Number of home visits by family support service, timeframe not reported | At least one psychosocial risk factor, are referred to early years support service, age <12 months | Machine learning - gradient boosted machine regression tree (number of visits) | None | Not reported | Continuous outcome | No | Not reported | Not reported | Not reported | High (4/4) |
| Ramsbottom2018  **Readmit predictor tool** | Readmission within 30 days | Discharged from a psychiatric inpatient unit, non-representative sample, age range not reported, USA | Additive (risk groups) | None | 25 | 2.9 | Algorithm | Not reported | Not reported | Not reported | High (3/4: Pa,A,O) |
| Yeguez 2020 | Step up in psychological treatment for anxiety in open-label trial |  | Logistic regression | None | 2 | 22.5 | No | Not reported | Not reported | Ratio True +:False + - 5:4 | High (2/4: O, A) |

Notes: model performance (AUC/calibration/classification) extracted for external validation sample where possible. Calibration-in-the-large would be 0 for a perfectly calibrated model, negative values suggest the model overestimates risk, while positive values suggest that the model underestimates risk. * indicates estimated maximum EPV because complete number of candidate predictors or events was not clearly reported. ** indicates that confidence intervals were not reported in original study. EPV = Events per (candidate) variable. Risk of bias: we present the overall risk of bias and number of domains with high risk. Pa = participants (study assessed as high risk of bias in participants domain of PROBAST), Pr = predictors, O = outcomes, A = analysis. AUC = area under the receiver operating characteristic curve, also known as c-index. PPV = positive predictive value. NPV = negative predictive value. Sens = sensitivity. Spec = specificity. BPD = bipolar disorder, BPD NOS = bipolar disorder, not otherwise specified.

## Table S2: Details of included validation studies

| Study ID, tool name | Outcome | Population | Model type  (model output) | Calibration | Discrimination, classification | Event number | ROB (domains) |
| --- | --- | --- | --- | --- | --- | --- | --- |
| JUSTICE |  |  |  |  |  |  |  |
| Barra 2018^1^  VRAG-R, J-SOAP-II, ERASOR | J-SOAP-II: sexual reoffending,  ERASOR: sexual reoffending  VRAG: general reoffending  Within 6 months | Male juvenile sexual offenders, age 12-18y | Additive/ structured professional judgement (total score, overall risk judgement) | Not reported | J-SOAP-II: AUC 0.74 (0.64 – 0.84)  ERASOR: AUC 0.76 (0.67 – 0.85)  VRAG: AUC 0.73 (0.68 – 0.78) | J-SOAP-II: 21  ERASOR: 21  VRAG: 129 | High: Pa, Pr, O, A |
| Cuervo 2018^2^  YLS/CMI-SRV | Reoffending (offending against people or property) within 2 years | Juveniles with a disciplinary record, age 14-17y | Additive (total score) | Not reported | AUC: 0.775 (0.72 – 0.83) | 99 | High: Pa, A |
| Hay 2018^3^  Residential Positive Achievement Scale (R-PACT) | Reoffending within 12 months. | Released juvenile offenders, age 10-20y | Not reported – proprietary scoring (risk groups, subscale total scores) | Not reported | Not reported for whole model | 1880 | High: Pa, A |
| Hutchins 2019^4^  PACTS | Reoffending (re-conviction or adult arrest) within 12 months | Juveniles under probation services, age 10-17y | Not reported (risk groups) | Not reported | AUC 0.561 (0.521 - 0.601) | 502 | High: Pa, A |
| Jeon 2020^5^  PCL:YV | Reoffending 9-18 months after release | Male probationers being released from residential probation facility, age 13-20y | Additive (total score) | Not reported | AUC 0.62 (0.49-0.76) (any recidivism) | 56 | High: Pr, O, A |
| Kaufman 2019^6^  SAVRY, STRESS | Reoffending (new court referral) within 12mo | Juveniles admitted to detention, age 12-18y | Checklist/summative models but tested in regression model (total score) | Not reported | Not reported | 213 | High: Pa, A |
| Kleeven 2020^7^  SAVRY, SAPROF-YV | Reoffending (conviction) - within 6 months | High-risk youth offenders with a history of violence, age 13-17. | Additive/SPJ (total score, and SPJ summary risk rating) | Not reported | SAVRY: AUC 0.66,  SAPROF-YV: AUC 0.74 | 30 | High: A |
| Koh 2020^8^  SAVRY, VRS-YV, SAPROF | Reoffending within 1 year | Male juvenile offenders sentenced to probation or juvenile detention, age 12-18y. | SAVRy: additive/SPJ (total risk scores) ,  VRS-yV: additive/SPJ (total risk score),  SAPROF: additive (total protective score) | Not reported | SAVRy: AUC 0.61 (any recidivism),  VRS-YV: AUC 0.57 (any recidivism),  SAPROF-YV: AUC 0.59 (any recidivism) | 36 (at 1 year) | High: 2/4: Pr, A |
| Krause 2021^9^  ERASOR, J-SOAP-II | Reoffending within 3 years | Male juvenile offenders convicted of a contact sexual offence, age 12-18y. Compare groups based on victim age. | ERASOR: SPJ (risk group),  J-SOAP-II: additive (total score) | Not reported | Not reported | 34 | High: Pr, O, A |
| Laurinavicius 2019^10^  Triarchic Pyschopathy measure (TriPM) | Misconduct (including any aggression, theft, substance use) reported by probation officer within 3 months | Male juvenile offenders under probation supervision, mean age 16 years. | Additive  (total score) | Not reported | AUC 0.63 (0.53 – 0.73) | 30 | High: O, A |
| Li 2020^11^  SAVRY | Reoffending, average follow-up 2 years | Asian Canadian and White Canadian youth on probation, age 13-20y. | Additive/SPJ (total score/risk groups) | Not reported | East/SE Asian Canadian: PPV 0.33, NPV 0.95, sens 0.75, spec 0.77  South Asian Canadian: PPV 0.39, NPV 1, sens 1, spec 0.68.  White Canadian: PPV 0.32, NPV 0.91, Sens 0.64, spec 0.73. | 118 (all groups) | High: A |
| Lockwood 2018^12^  YLS/CMI | Reoffending within 2 years. | Youth undergoing court-ordered assessment for sentencing purposes, mean age 15. | Additive (total score) | Not reported | AUC: 0.68 (0.53 – 0.83) | 48 | High: Pa, A |
| McCuish 2018^13^  PCL:YV | Violent reoffending (convictions) in emerging adulthood (age 18-23) | Youth in open and secure custody for juvenile offenders, age 12-19y. | Additive  (total score) | Not reported | White: AUC 0.52 (0.45 – 0.60)  Indigenous: AUC 0.66 (0.56 – 0.76) | White youth: 116  Indigenous youth: 71 | High: A |
| McKenzie 2019^14^  PACT | Reoffending (re-arrest or re-referral) within 12 months | Juveniles referred to a probation department, age 10-18y | Additive, and risk matrix (risk groups) | Not reported | AUC: 0.62 (0.56 – 0.68) | 77 | High: Pa, A |
| Muir 2020^15^  SAVRY | Reoffending, mean follow-up 1.96 years | Indigenous and White Canadian juvenile offenders, age 12-20y | Additive/SPJ (total score) | Not reported | Indigenous females PPV 0.52 (0.33-0.70), NPV 0.87 (0.60-0.98), sens 0.89 (0.65-0.98), spec 0.46 (0.28-0.66) (for violent recidivism)  Indigenous Males PPV 0.51 (0.38-0.64), NPV 0.85 (0.75-0.92),sens 0.74 (0.58-0.86), spec 0.68 (0.57-0.77) (for violent recidivism) | 253 (all groups violent recidivism) | High: A |
| Ortega-Campos 2020^16^  SAVRY, YLS/CMI | Reoffending (new legal case) within 2 years | Offenders processed through juvenile court, mean age 15.6y | Additive (total score) | Not reported | SAVRy: AUC 0.745,  YLS/CMI: AUC 0.757 | 211 | High: Pr, A |
| Papp 2019^17^  YLS/CMI | Reoffending within 2 years | Youth in contact with juvenile court for delinquency or truancy, age 9-18y | Additive (total score, risk groups) | Not reported | AUC 0.59 (0.56 – 0.62) | 627 | High: Pa, A |
| Rasmussen 2018^18^  JSORRAT, MEGA | Sexual reoffending, mean time to outcome 15 months. | Juvenile offenders in secure residential facility, age 10-19. | Additive  (total score, risk groups) | No sig. Difference in expected vs observed probability for MEGA. | MEGA: AUC 0.67 (0.52 – 0.82)  JSORRAT: AUC 0.57 (0.43 – 0.72) | 21 | High: Pa, O, A |
| Rojas 2019^19^  VRS-YSO  (compared to J-SOAP-II, ERASOR, J-SORRAT-II) | Sexual reoffending (re-conviction) mean follow up 11 y. | Male sexual offenders, mean age 14. | Additive (total score) | Not reported | VRS-YSO: AUC 0.77 (0.64 – 0.91)  J-SOAP-II: AUC 0.69 (0.54 - .89)  ERASOR: AUC 0.67 (0.50 – 0.83)  J-SORRAT-II: AUC 0.76 (0.55 – 0.96) | 39 | High: Pa, A |
| Rojas 2021^20^  PCL:YV | Reoffending, timing unclear | Youth sexual offenders receiving mental health services, age 12-19y | Additive (total score) | Not reported | General recidivism: AUC 0.67 (0.55-0.78) | 39 | High: A |
| Schwartz-Mette 2019^21^  J-SOAP-II | Adult reoffending (sexual/violent/general) | Juvenile sexual offenders, male, excludes those who recidivate as juveniles, age <18y | Additive (total score) | Not reported | AUC: 0.76 (0.56 – 0.97) | 6 | High: Pa, Pr, A |
| Schwartz-Mette 2020^22^  J-SOAP-II | Reoffending (new charge) follow-up range 7.6-15.5 months | Juveniles charged with contact sexual offences referred to forensic services, age 10-20y. | Additive (total score) | Not reported | Sexual offending: AUC 0.76 (0.56 - 0.97). | 6 | High: Pr, A |
| Viljoen 2018^23^  SAVRY (protective total score), YLS/CMI (strengths total score) | SAVRY: violent reoffending  YLS/CMS: general reoffending (charges) within 2 years | Youth on probation, any offence type, age 12-18y | Additive (total score) | Not reported | SAVRY: AUC 0.62 (0.52 – 0.72)  YLS/CMI: AUC 0.60 (0.50 – 0.70) | 31 | High: Pr, A |
| Vincent 2019^24^  YLS/CMI, SAVRY, HCR20, VRAG | Reoffending, follow-up range 1-35 years | Offenders with history of delinquency offences. Age groups: 11-16y, 16-24y, >24y | Additive/ Structured Professional Judgement (total score, risk groups) | Not reported | SAVRY: AUC 0.72 (0.65 – 0.79)  YLS/CMI: AUC 0.70 (0.65 – 0.75) | SAVRY: 65  YLS/CMI: 115 | High: Pa, Pr, O, A |
| Vitopoulos 2019^25^  YLS/CMI | Reoffending (re-conviction) within 2 years | Youth ordered to attend a juvenile justice clinic, age 13-19y | Additive scale, incremental value tested in regression model (risk groups, total score) | Not reported | AUC: 0.59 (0.48 – 0.70) | 49 | High: Pa, A |
| Wijetunga 2018^26^  J-SOAP-II | Sexual reoffending (re-arrest), mean time to outcome 63.7 months | Juvenile sexual offenders discharged from correctional facility or treatment programme. Age 14-19y. | Additive  (total score) | Not reported | 14-16y: AUC 0.75 (0.58 – 0.92)  17-19y: AUC 0.58 (0.37 – 0.79) | 14-16y: 5  17-19y: 8 | High: Pa, A. Unclear: Pr |
| FUTURE DIAGNOSIS |  |  |  |  |  |  |  |
| Brathwaite 2021^27^  Pelotas model | Presence of clinically relevant depression at age 18+ | Child soldiers and war-affected civilians in Nepal, age 11-18y. | Penalized logistic regression (% risk) | Cal-in-large 0.00, cal slope 1.50 | AUC: 0.83 (0.74-0.91) (refitted model) | 25 | High (1/4, A) |
| Brathwaite 2020^28^  Pelotas model | Depression diagnosis after 3 years | School-attending children in Nigeria age 14-16y | Penalized logistic regression (% risk) | Cal-in-large: 0.00 Cal slope 1.07 | AUC: 0.66 (0.63-0.70) [fully refitted] | Approx 228 | Low |
| Hadders‐Algra 2019^29^  Standardized Infant Neuro-Developmental Assessment (SINDA) | Behavioural disorders (including ASD/ADHD) at age 24-57 months | Infants at high risk of neurodevelopmental disorder seen in specialist paediatric clinic. Age 6 weeks – 12 months | Additive (binary) | Not reported | Sens 0.52  Spec 0.96 | 25 | High: Pa, O, A |
| Raza 2019^30^  Short Quantitative Checklist for Autism in toddlers (Q-CHAT-10) | ASD or atypical development at 36 months old | Siblings of children with ASD, high-risk sample. Age 18-24 months | Additive (binary) | Not reported | Sens: 0.75  Spec: 0.63 | 25 | High: A |
| Raza 2019b^31^  Infant-Toddler Social Emotional Assessment (ITSEA) | ASD at 36 months old | Siblings of children with ASD and controls, high-risk group. Age 18 months | Additive, scale domains analysed separately (binary/ subscale total scores) | Not reported | Not reported for full model | 93 | High: A |
| Usta 2020^32^  Child Behaviour Checklist (CBCL), Brief Infant and Toddler Emotional Assessment (BITSEA) | Psychiatric diagnosis in adolescence, 10 years after prediction | Non-random sample evaluated by child psychiatry residents, age 1-3y | Additive (subscale scores) | Not reported | CBCL withdrawn/depressed scale: AUC 0.72 | 35 | High: Pa, O, A |
| CHILD PROTECTION |  |  |  |  |  |  |  |
| de Ruiter 2019^33^  Child Abuse Risk Evaluation - Dutch version (CARE-NL) | Out-of-home placement or placement under court supervision (as proxy for abuse recurrence) within 3 years | Family reported to child protection service and fully investigated. Community sample. Age 0-17y | Additive/structured professional judgement (risk groups, total score) | Not reported | Total score AUC 0.60  SPJ AUC: 0.78 | Not reported | High: Pa, Pr, O, A |
| Kaye 2019^34^  Family Needs screener (FNS) | Family violence (child maltreatment or IPV). Time to outcome not clear. | Army families with child <3y/pregnant/awaiting adoption. | Additive, plus individual “high-risk” variables (risk group, binary) | Not reported | Sens: 0.76  Spec: 0.97 | 1148 | High: Pa, O, A |
| Kelly 2019^35^  Unnamed  Incremental validity study testing previously developed model for novel outcome, with extra predictors. | Abusive head trauma age 0-2y | Cases vs general pop controls. Neonates. | Logistic regression (probability) | Not reported | AUC: 0.91 (0.88 – 0.94) | 142 | High: Pa, Pr, O, A |
| Logan-Greene 2018^36^  Structured decision making (SDM) | Chronic neglect (≥4 reports of neglect) within 4 years | Nested case control of children referred to child protective services with initial report of neglect. Age range not reported. | Not reported (total score, risk groups) | Not reported | Overall classification accuracy: 0.79 | 430 | High: Pa, A, Unclear: O |
| Schols 2019^37^  Early Risk of Physical Abuse and Neglect Sclae (ERPANS) | Report to child welfare services within 22 months +/- involvement of authorities or consideration of intervention for parents | Community cohort of parents of newborns. | Regression used to test factors from original additive model (output type not reported) | Not reported | Not reported | 70 | High: O, A |
| Vaithianathan 2020^38^  Alleghany Family Screening Tool (AFST) | Hospitalization or ED visit with injury, timing unclear | Referrals to child protection services for alleged abuse or neglect, mean age 8y. | LASSO regression (risk ventiles) | Not reported | Not reported | All-cause injury: 8309 | High: Pr, O, A |
| SELF-HARM |  |  |  |  |  |  |  |
| Casiano 2019^39^  Inmate Security Assessment (ISA) | Self-harm during detainment, time to outcome not reported | Youth in custody in Manitoba, age 12-18y | Not reported (risk groups) | Not reported | AUC: 0.72 (0.69 – 0.75)  Sens: 0.03, Spec 0.99 | 240 | High: Pa, O, A |
| DeVylder 2019^40^  Ask Suicide-screening Questionnaire (ASQ) | Repeat self-harm with attendance to ED within 3 months | Individuals presenting to ED, enriched for those presenting with behavioural/psychiatric complaint, age 8-18y | Checklist (binary) | Not reported | Sens: 0.765 (68.4-83.3)  Spec: 0.851 (84.5-85.7)  PPV: 0.049  NPV: 0.997 | 136 | High: Pa, O, A |
| Evans 2019^41^  Functional Analysis in Care Environments - Child and Adolescent Risk Assessment Suite (FACE-CARAS) | Self-harm within 6 months.  Other outcomes reported: violence, suicidal behaviour, self-neglect, accident, abuse | Children with case open for >6 months to community CAMHS service. Age 5-18y | Structured Professional judgement (total score, binary) | Not reported | AUC: 0.80 (0.71 – 0.86)  Sens: 0.50,  Spec: 0.91 | 34 | High: Pa, O, A |
| Steeg 2018^42^  Manchester Self-harm rule, react self-harm rule, SAD PERSONS scale, Modified sad persons scale (MSHR, react, SPS, MSPS) | Repeat self-harm with attendance to hospital within 6 months | Individuals presenting to ED with self-harm, age <19 | Additive (risk groups, total score, binary) | Not reported | MSHR: AUC 0.73 (0.68 – 0.78)  React: AUC 0.73 (0.68 – 0.78)  SPS: AUC 0.47 (0.42 – 0.52)  MSPS: AUC 0.48 (0.42 - 0.53) | Not reported (for age group <19) | High: Pr, O, A |
| SYMPTOMS/FUNCTIONAL OUTCOMES | |  |  |  |  |  |  |
| Garber 2018^43^  Unnamed | Days to depression onset/depression-free days | Community sample of youth at risk of depression (current subsyndromal depression/prior depression and parent with depressive disorder. Age 13-17y | Decision tree (risk groups) | Not reported | Not reported | 135* | High: Pa, O. Unclear: O |
| Stanfield 2019^44^  Structural Interview for Schizotypy, Childhood behavioural checklist, and combined (SIS, CBCL) | Psychotic symptoms | Community sample with special educational needs. Age 13-22y | Risk matrix (risk groups, binary) | Not reported | Sens: 0.67  Spec: 0.99 | 9 | High: pa, O, A |
| SUBSTANCE USE |  |  |  |  |  |  |  |
| Linakis 2019^45^  National Institute on Alcohol Abuse and Alcoholism 2-question screen (NIAAA) | Number of drinking days and alcohol use disorders at 1 year | Adolescents presenting to paediatric emergency department. Age 12-17y | Risk matrix (risk groups) | Not reported | AUC: 0.862  Sens: 0.86  Spec: 0.78 | 29 | High: A, Unclear: O |
| Menon 2019^46^  Child Behaviour Checklist (CBCL), Youth Self-Report (YSR), CRAFFT | Substance use within 36 months | Families being investigated for child maltreatment, community sample. | Decision tree (probability) | Hosmer-Lemeshow χ^2^ female: 7.8, male: 15.79 | Females: AUC 0.82  Males: AUC 0.72 | 280 | High: A, Unclear: O |
| Seo 2020^47^  Unnamed | Onset of e-cigarette use within 12 months | Population cohort, age 12-17y | Checklist (high/low risk groups) | Not reported | Spec 0.73  Sens 0.57 | 988 | High: A |
| Shadel 2019^48^  National Institutes on Alcohol and Alcoholism screening guide (NIAAA SG) | Cigarette smoking within 6 months. | Primary care cohort, enriched for high risk. Prospective observational and treatment studies. USA. Age 12-18y | Risk matrix (risk groups) | Not reported | Sens: 0.62  Spec: 0.70 | Not reported | High: Pa, O, A |

Notes: *: event number is an estimate, actual number not reported. ERASOR = Estimate of Risk of Adolescent Sexual Offense Recidivism, J-SOAP-II = Juvenile Sex Offender Assessment Protocol-II, JSORRAT-II = Juvenile Sexual Offense Recidivism Risk Assessment Tool, MEGA = Multiplex Empirically Guided Inventory of Ecological Aggregates for Assessing Sexually Abusive Adolescents and Children, PACT = Positive Achievement Change Tool, PCL:YV = Psychopathy Checklist:Youth Version, SAVRY = Structured Assessment of Violence Risk in Youth, SAPROF - YV = Structured Assessment of Protective Factors - youth Version, STRESS = Structured Trauma-Related Experience and Symptoms, VRAG-R = Violence Risk Appraisal Guide - Revised, VRS-YV = Violence risk score – Youth Version, YLS/CMI = Youth Level of Service/Case Management Inventory. AUC = area under the curve, also known as c-statistic. Sens = sensitivity. Spec = specificity. Risk of bias (ROB) assessed with the PROBAST checklist in four domains: Participants (Pa), Predictors (Pr), Outcome (O) and Analysis (A). Overall risk of bias is High if any one domain has high risk of bias.

## Table S3: compliance with reporting and methodology recommendations for included model development studies

|  |  | **Reporting: methods** | | | | | | **Methodology** | | | **Reporting: results** | | | |
| --- | --- | --- | --- | --- | --- | --- | --- | --- | --- | --- | --- | --- | --- | --- |
| **Outcome type** | **Author and Year** | **Eligibility** | **Age range** | **Predictors** | **Outcome** | **Missing data** | **Modelling methods** | **EPV ≥10 (main model)** | **Internal validation** | **External validation** | **Full model reported** | **Simplified/online calculator** | **Report Discrimination** | **Report Calibration** |
| **Justice** | **Barnes 2018**^49^ | ✓ |  | ✓ | ✓ |  |  |  |  |  | ✓ |  | ✓ |  |
|  | **Choi 2020**^50^ | ✓ | ✓ |  | ✓ |  |  | ✓ | ✓ |  |  | ✓ | ✓ |  |
|  | **Kang 2019**^51^ ***** | ✓ | ✓ |  |  |  |  |  |  |  |  |  |  |  |
|  | **McKay 2019**^52^ | ✓ | ✓ |  |  |  | ✓ |  | ✓ |  | ✓ | ✓ | ✓ |  |
|  | **Norris 2018**^53^ | ✓ | ✓ | ✓ |  |  |  |  |  |  | ✓ | ✓ | ✓ |  |
|  | **Ozkan 2019**^54^ | ✓ |  |  |  |  |  |  | ✓ |  |  |  | ✓ |  |
|  | **Ting 2018**^55^ | ✓ | ✓ |  |  |  | ✓ |  | ✓ |  |  |  | ✓ |  |
|  | **Van der Put 2020**^56^ | ✓ | ✓ |  | ✓ |  | ✓ |  | ✓ |  | ✓ | ✓ | ✓ |  |
|  | **Villanueva 2019**^57^ | ✓ | ✓ |  | ✓ | ✓ |  |  |  |  | ✓ |  | ✓ |  |
| **Future diagnosis** | **Birmaher 2018**^58^ | ✓ | ✓ |  | ✓ | ✓ |  |  | ✓ | ✓ |  | ✓ | ✓ |  |
|  | **Caye 2018**^59^ | ✓ |  | ✓ | ✓ | ✓ |  | ✓ | ✓ | ✓ |  | ✓ | ✓ | ✓ |
|  | **Cohen 2019**^60^ | ✓ | ✓ | ✓ | ✓ | ✓ |  |  | ✓ | ✓ | ✓ |  | ✓ | ✓ |
|  | **Hamasaki 2020**^61^ | ✓ | ✓ | ✓ | ✓ |  |  |  |  |  |  | ✓ | ✓ | ✓ |
|  | **Hankin 2018**^62^ | ✓ | ✓ | ✓ | ✓ |  |  | ✓ |  |  | ✓ |  |  |  |
|  | **Koning 2019**^63^ | ✓ | ✓ | ✓ | ✓ | ✓ | ✓ | ✓ | ✓ |  | ✓ |  | ✓ | ✓ |
|  | **Lewis 2019**^64^ | ✓ | ✓ | ✓ | ✓ | ✓ |  | ✓ | ✓ |  | ✓ |  | ✓ | ✓ |
|  | **Malda 2019**^65^ | ✓ | ✓ | ✓ | ✓ | ✓ |  | ✓ | ✓ |  | ✓ |  | ✓ | ✓ |
|  | **Moore 2021**^66^ | ✓ | ✓ | ✓ | ✓ |  | ✓ |  |  |  |  | ✓ | ✓ |  |
|  | **Meehan 2020**^67^ | ✓ | ✓ | ✓ | ✓ | ✓ |  | ✓ | ✓ |  | ✓ |  | ✓ | ✓ |
|  | **Nichols 2018**^68^ | ✓ | ✓ |  | ✓ | ✓ |  | ✓ | ✓ |  |  |  | ✓ |  |
|  | **Pijl 2019**^69^ | ✓ | ✓ | ✓ | ✓ | ✓ |  |  |  |  | ✓ |  | ✓ |  |
|  | **Rabelo-da-Ponte 2020**^70^ | ✓ | ✓ |  | ✓ | ✓ | ✓ | ✓ | ✓ |  |  |  | ✓ |  |
|  | **Rahman 2020**^71^ | ✓ |  |  |  | ✓ | ✓ |  | ✓ |  |  |  | ✓ |  |
|  | **Rocha 2021**^72^ | ✓ | ✓ | ✓ | ✓ | ✓ | ✓ |  | ✓ | ✓ | ✓ |  | ✓ | ✓ |
|  | **Sacrey2018**^73^ | ✓ | ✓ |  | ✓ | ✓ |  |  |  |  | ✓ | ✓ | ✓ |  |
|  | **Tate 2020**^74^ | ✓ |  | ✓ |  | ✓ | ✓ |  | ✓ |  |  |  | ✓ |  |
|  | **Van Meter 2021**^75^ |  |  | ✓ |  |  | ✓ | ✓ | ✓ | ✓ |  |  | ✓ |  |
| **Child protection** | **Elgin 2018**^76^ | ✓ | ✓ |  | ✓ | ✓ | ✓ | ✓ | ✓ |  |  |  | ✓ |  |
|  | **Hansmann 2020**^77^ | ✓ | ✓ | ✓ | ✓ | ✓ | ✓ | - | - | - | ✓ | - | ✓ | ✓ |
|  | **Thurston 2018**^78^ | ✓ |  |  |  |  |  | ✓ |  |  | ✓ |  |  |  |
|  | **Vaithianathan 2018**^79^ | ✓ | ✓ | ✓ | ✓ |  |  |  |  |  | ✓ |  |  |  |
|  | **Walsh 2020**^80^ | ✓ | ✓ | ✓ | ✓ |  |  |  | ✓ |  |  |  | ✓ |  |
| **Self-harm/ suicide** | **Hill 2019**^81^ | ✓ | ✓ | ✓ | ✓ | ✓ |  |  |  |  | ✓ |  |  |  |
|  | **King 2019**^82^ | ✓ | ✓ | ✓ | ✓ | ✓ |  |  | ✓ |  | ✓ |  | ✓ |  |
|  | **Pettit 2018**^83^ ***** |  |  |  |  |  |  |  |  |  |  | ✓ |  |  |
|  | **Simon 2018**^84^ | ✓ | ✓ | ✓ | ✓ |  |  | ✓ | ✓ |  | ✓ |  | ✓ | ✓ |
|  | **Su 2020**^85^ | ✓ | ✓ |  | ✓ |  |  |  | ✓ |  |  |  | ✓ |  |
|  | **Walsh 2018**^86^ | ✓ |  |  | ✓ | ✓ |  |  | ✓ |  |  |  | ✓ | ✓ |
| **Symptoms and functional outcomes** | **Birmaher 2020**^87^ | ✓ |  | ✓ | ✓ |  | ✓ |  | ✓ |  |  | ✓ | ✓ | ✓ |
|  | **Goodwin 2019**^88^ | ✓ | ✓ | ✓ | ✓ | ✓ |  |  | ✓ |  |  |  | ✓ |  |
|  | **Latham 2019**^89^ | ✓ | ✓ | ✓ | ✓ | ✓ | ✓ | ✓ | ✓ |  | ✓ |  | ✓ | ✓ |
|  | **Li 2019**^90^ | ✓ | ✓ |  | ✓ | ✓ |  |  | ✓ |  | ✓ | ✓ | ✓ | ✓ |
|  | **Shui 2018**^91^ | ✓ | ✓ |  | ✓ | ✓ |  |  | ✓ |  |  |  |  |  |
|  | **VanMinde 2019**^92^ ***** | ✓ | ✓ |  |  |  |  |  |  |  | ✓ | ✓ |  |  |
| **Substance misuse** | **Afzali 2018**^93^ | ✓ |  | ✓ | ✓ | ✓ |  | ✓ | ✓ | ✓ |  |  | ✓ |  |
|  | **Sylvestre 2018**^94^ | ✓ | ✓ | ✓ | ✓ | ✓ |  |  | ✓ |  |  | ✓ | ✓ | ✓ |
|  | **Zhang-James 2020**^95^ | ✓ | ✓ | ✓ | ✓ | ✓ | ✓ |  | ✓ |  |  |  | ✓ | ✓ |
| **Other** | **Evers 2018**^96^ | ✓ | ✓ |  |  | ✓ |  |  |  |  | ✓ |  |  |  |
|  | **Grebla 2019**^97^ | ✓ | ✓ |  | ✓ |  |  |  | ✓ |  |  |  |  |  |
|  | **Ogrim 2019**^98^ | ✓ | ✓ |  | ✓ |  |  |  |  |  | ✓ |  | ✓ |  |
|  | **Ramsbottom 2018**^99^ |  |  |  |  |  |  |  |  |  | ✓ | ✓ |  |  |
|  | **Yeguez 2020**^100^ |  |  |  |  |  |  | ✓ |  |  |  |  |  |  |

## Note: ✓ indicates that full details are reported (as per TRIPOD guidance) or that key methodological recommendations were reported as met. * = tool developed without data (eg. based on expert opinion)

## References

1 Barra S, Bessler C, Landolt MA, Aebi M. Testing the validity of criminal risk assessment tools in sexually abusive youth. *Psychol Assess* 2018; **30**: 1430–43.

2 Cuervo K, Villanueva LL. Prediction of recidivism with the Youth Level of Service/Case Management Inventory (Reduced Version) in a sample of young Spanish offenders. *Int J Offender Ther Comp Criminol* 2018; **62**: 3562–80.

3 Hay C, Widdowson AO, Bates M, Baglivio MT, Jackowski K, Greenwald MA. Predicting recidivism among released juvenile offenders in Florida. *Youth Violence Juv Justice* 2018; **16**: 97–116.

4 Hutchins EP. Assessing the predictive utility of the Positive Achievement Change Tool at a Texas juvenile justice agency. 2019; published online Jan. https://digitalcommons.utep.edu/open_etd/91 (accessed June 1, 2021).

5 Jeon H, Boccaccini MT, Jo E, Jang H, Murrie DC. Rater experience and the predictive validity of Psychopathy Checklist: Youth Version scores. *Psychiatry Psychol Law* 2020; **27**: 912–23.

6 Kaufman H. Trauma as a responsivity factor among detained adolescents using the structured-trauma related experience and symptoms screener (STRESS). *Diss Abstr Int Sect B Sci Eng* 2019; **80**: No-Specified.

7 Kleeven ATH, de Vries Robbé M, Mulder EA, Popma A. Risk assessment in juvenile and young adult offenders: predictive validity of the SAVRY and SAPROF-YV. *Assessment* 2020; : 107319112095974.

8 Koh LL, Day A, Klettke B, Daffern M, Chu CM. The predictive validity of three youth violence assessment instruments: the SAVRY, VRS-YV, and SAPROF-YV. *Int J Offender Ther Comp Criminol* 2020; : 0306624X2097088.

9 Krause C, Roth A, Landolt MA, Bessler C, Aebi M. Validity of risk assessment instruments among juveniles who sexually offended: victim age matters. *Sex Abuse* 2021; **33**: 379–405.

10 Laurinavicius A, Sellbom M, Klimukiene V, *et al.* Examination of triarchic psychopathy measure in a sample of Lithuanian juvenile offenders. *Psychol Assess* 2019; **32**: 407–13.

11 Li SMY, Viljoen JL, Christiansen AK, Muir NM. Predictive validity of the Structured Assessment of Violence Risk in Youth (SAVRY) among a sample of Asian Canadian youth on probation. *Law Hum Behav* 2020; **44**: 485–501.

12 Lockwood I, Peterson-Badali M, Schmidt F. The relationship between risk, criminogenic need, and recidivism for Indigenous justice-involved youth. *Crim Justice Behav* 2018; **45**: 1688–708.

13 McCuish EC, Mathesius JR, Lussier P, Corrado RRAI. The cross-cultural generalizability of the Psychopathy Checklist: Youth Version for adjudicated indigenous youth. *Psychol Assess* 2018; **30**: 192–203.

14 McKenzie SM. A validation study of the Positive Achievement Change Tool (PACT). *Diss Abstr Int Sect B Sci Eng* 2019; **80**: No-Specified.

15 Muir NM, Viljoen JL, Jonnson MR, Cochrane DM, Rogers BJ. Predictive validity of the Structured Assessment of Violence Risk in Youth (SAVRY) with Indigenous and Caucasian female and male adolescents on probation. *Psychol Assess* 2020; **32**: 594–607.

16 Ortega-Campos E, De la Fuente-Sánchez L, Zaldívar-Basurto F. Predicting risk of recidivism in Spanish young offenders: Comparative analysis of the SAVRY and YLS/CMI. *Psicothema* 2020; : 221–8.

17 Papp J, Campbell CA, Anderson VR. Assessing the incremental validity of Andrews and Bonta’s “moderate four” predictors of recidivism using a diverse sample of offending and truant youth. *Int J Offender Ther Comp Criminol* 2019; **63**: 854–73.

18 Rasmussen LAL. Comparing predictive validity of JSORRAT-II and MEGA(music note) with sexually abusive youth in long-term residential custody. *Int J Offender Ther Comp Criminol* 2018; **62**: 2937–53.

19 Rojas EY, Olver ME. Validity and reliability of the Violence Risk Scale–Youth Sexual Offense Version. *Sex Abuse* 2019; : 107906321985806.

20 Rojas EY, Olver ME. Juvenile psychopathy and community treatment response in youth adjudicated for sexual offenses. *Int J Offender Ther Comp Criminol* 2021; : 0306624X2199406.

21 Schwartz-Mette RA, Righthand S, Hecker J, Dore G, Huff R. Long-term predictive validity of the Juvenile Sex Offender Assessment Protocol–II: research and practice implications. *Sex Abuse* 2019; : 107906321982587.

22 Schwartz-Mette RA, Righthand S, Hecker J, Dore G, Huff R. Long-term predictive validity of the Juvenile Sex Offender Assessment Protocol–II: research and practice implications. *Sex Abuse* 2020; **32**: 499–520.

23 Viljoen JL, Bhanwer AK, Shaffer CS, Douglas KS. Assessing protective factors for adolescent offending: a conceptually informed examination of the SAVRY and YLS/CMI. *Assessment* 2018; : 107319111876843.

24 Vincent GM, Drawbridge D, Davis M. The validity of risk assessment instruments for transition-age youth. *J Consult Clin Psychol* 2019; **87**: 171–83.

25 Vitopoulos NA, Peterson-Badali M, Brown S, Skilling TA. The relationship between trauma, recidivism risk, and reoffending in male and female juvenile offenders. *J Child Adolesc Trauma* 2019; **12**: 351–64.

26 Wijetunga C, Martinez R, Rosenfeld B, Cruise K. The influence of age and sexual drive on the predictive validity of the Juvenile Sex Offender Assessment Protocol–Revised. *Int J Offender Ther Comp Criminol* 2018; **62**: 150–69.

27 Brathwaite R, Rocha TB-M, Kieling C, *et al.* Predicting the risk of depression among adolescents in Nepal using a model developed in Brazil: the IDEA Project. *Eur Child Adolesc Psychiatry* 2021; **30**: 213–23.

28 Brathwaite R, Rocha TB-M, Kieling C, *et al.* Predicting the risk of future depression among school-attending adolescents in Nigeria using a model developed in Brazil. *Psychiatry Res* 2020; **294**: 113511.

29 Hadders‐Algra M, Tacke U, Pietz J, Rupp A, Philippi H. Standardized Infant NeuroDevelopmental Assessment developmental and socio‐emotional scales: reliability and predictive value in an at‐risk population. *Dev Med Child Neurol* 2019; : dmcn.14423.

30 Raza S, Zwaigenbaum L, Sacrey L-AR, *et al.* Brief report: evaluation of the Short Quantitative Checklist for Autism in Toddlers (Q-CHAT-10) as a brief screen for autism spectrum disorder in a high-risk sibling cohort. *J Autism Dev Disord* 2019; **49**: 2210–8.

31 Raza S, Sacrey L-AR, Zwaigenbaum L, *et al.* Relationship between early social-emotional behavior and autism spectrum disorder: A high-risk sibling study. *J Autism Dev Disord* 2019; published online March. DOI:10.1007/s10803-019-03977-3.

32 Usta MB, Karabeki̇Roglu K, Aydin M, *et al.* The predictive social and emotional measures in toddlerhood for psychiatric morbidity in adolescence. *Konuralp Tıp Derg* 2020; : 66–72.

33 de Ruiter C, Hildebrand M, van der Hoorn S. The Child Abuse Risk Evaluation Dutch Version (CARE-NL): A retrospective validation study. *J Child Custody* 2019; : 1–21.

34 Kaye MP, Saathoff-Wells T, Ferrara AM, Morgan NR, Perkins DF. Exploratory structural equation modeling analysis and validity of the Family Needs Screener. *J Interpers Violence* 2019; : 088626051988851.

35 Kelly P, Thompson JMD, Rungan S, *et al.* Do data from child protective services and the police enhance modelling of perinatal risk for paediatric abusive head trauma? A retrospective case-control study. *BMJ Open* 2019; **9**: e024199.

36 Logan-Greene P, Semanchin Jones A. Predicting chronic neglect: Understanding risk and protective factors for CPS-involved families. *Child Fam Soc Work* 2018; **23**: 264–72.

37 Schols MWA, Serie CMB, Broers NJ, de Ruiter C. Factor analysis and predictive validity of the Early Risks of Physical Abuse and Neglect Scale (ERPANS): A prospective study in Dutch public youth healthcare. *Child Abuse Negl* 2019; **88**: 71–83.

38 Vaithianathan R, Putnam-Hornstein E, Chouldechova A, Benavides-Prado D, Berger R. Hospital injury encounters of children identified by a predictive risk model for screening child maltreatment referrals: evidence from the Allegheny Family Screening Tool. *JAMA Pediatr* 2020; **174**: e202770.

39 Casiano H, Bolton S-L, Katz LY, Bolton JM, Sareen J. Evaluation of a suicide risk assessment tool in a large sample of detained youth. *J Can Acad Child Adolesc Psychiatry J Acad Can Psychiatr Enfant Adolesc* 2019; **28**: 105–14.

40 DeVylder JE, Ryan TC, Cwik M, *et al.* Assessment of selective and universal screening for suicide risk in a pediatric Emergency Department. *JAMA Netw Open* 2019; **2**: e1914070–e1914070.

41 Evans SA, Young D, Tiffin PA. Predictive validity and interrater reliability of the FACE-CARAS toolkit in a CAMHS setting. *Crim Behav Ment Health* 2019; **29**: 47–56.

42 Steeg S, Quinlivan L, Nowland R, *et al.* Accuracy of risk scales for predicting repeat self-harm and suicide: A multicentre, population-level cohort study using routine clinical data. *BMC Psychiatry* 2018; **18**: 113.

43 Garber J, Weersing VR, Hollon SD, *et al.* Prevention of depression in at-risk adolescents: moderators of long-term response. *Prev Sci* 2018; **19**: 6–15.

44 Stanfield AC, McKechanie AG, Lawrie SM, Johnstone EC, Owens DGC. Predictors of psychotic symptoms among young people with special educational needs. *Br J Psychiatry* 2019; **215**: 422–7.

45 Linakis JG, Bromberg JR, Casper TC, *et al.* Predictive validity of a 2-question alcohol screen at 1-, 2-, and 3-year follow-up. *Pediatrics* 2019; **143**: e20182001.

46 Menon SV, Thakur H, Shorey RC, Cohen JR. Predicting adolescent substance use in a child welfare sample: a multi-indicator algorithm. *Assessment* 2019; : 107319111988096.

47 Seo D-C, Kwon E, Lee S, Seo J. Using susceptibility measures to prospectively predict ever use of electronic cigarettes among adolescents. *Prev Med* 2020; **130**: 105896.

48 Shadel WG, Seelam R, Parast L, Meredith LS, D’Amico EJ. Is the National Institute on Alcohol Abuse and Alcoholism screening guide useful for identifying adolescents at risk for later cigarette smoking? A prospective study in primary care clinics. *J Addict Med* 2019; **13**: 119–22.

49 Barnes AR. An assessment of protective factors in predicting juvenile reoffending. *Diss Abstr Int Sect Humanit Soc Sci* 2018; **78**: No-Specified.

50 Choi E. Enhancing the integrated treatment assessment for youth sexual recidivism prediction: toward a Washington state-unified risk assessment system. 2020; published online May. https://www.proquest.com/openview/d9e7c0d11539f52c28e5f976fff42431/1?pq-origsite=gscholar&cbl=18750&diss=y (accessed May 29, 2021).

51 Kang T, Beltrani A, Manheim M, *et al.* Development of a risk/treatment needs and progress protocol for juveniles with sex offenses. *Transl Issues Psychol Sci* 2019; **5**: 154–69.

52 McKay PD. Decisions leading to inequalities in the juvenile justice system: using data to predict delinquent outcomes, inform decisions and reduce disparities for justice-involved youth. 2019; published online May. https://scholarworks.umt.edu/etd/11378/ (accessed May 29, 2021).

53 Norris G, Griffith G, West M. Validation of the Ceredigion Youth Screening Tool. *Int J Offender Ther Comp Criminol* 2018; **62**: 3727–45.

54 Ozkan T, Clipper SJ, Piquero AR, Baglivio M, Wolff K. Predicting sexual recidivism. *Sex Abuse* 2019; **32**: 375–99.

55 Ting MH, Chu CM, Zeng G, Li D, Chng GS. Predicting recidivism among youth offenders: Augmenting professional judgement with machine learning algorithms. *J Soc Work* 2018; **18**: 631–49.

56 van der Put CE. The development of a risk and needs assessment instrument for truancy. *Child Youth Serv Rev* 2020; **109**: 104721.

57 Villanueva LL, Basto-Pereira M, Cuervo K. How to improve the YLS/CMI? exploring a particularly predictive combination of items. *Int J Offender Ther Comp Criminol* 2019; : 0306624X1988192.

58 Birmaher B, Merranko JA, Goldstein TR, *et al.* A risk calculator to predict the individual risk of conversion from subthreshold bipolar symptoms to bipolar disorder I or II in youth. *J Am Acad Child Adolesc Psychiatry* 2018; **57**: 755-763.e4.

59 Caye A, Agnew-Blais J, Arseneault L, *et al.* A risk calculator to predict adult attention-deficit/hyperactivity disorder: generation and external validation in three birth cohorts and one clinical sample. *Epidemiol Psychiatr Sci* 2020; **29**: e37.

60 Cohen JR, Thakur H, Young JF, Hankin BL. The development and validation of an algorithm to predict future depression onset in unselected youth. *Psychol Med* 2019; : 1–9.

61 Hamasaki Y, Nakayama T, Hikida T, Murai T. Combined pattern of childhood psycho-behavioral characteristics in patients with schizophrenia: a retrospective study in Japan. *BMC Psychiatry* 2021; **21**: 57.

62 Hankin BL, Young JF, Gallop R, Garber J. Cognitive and interpersonal vulnerabilities to adolescent depression: classification of risk profiles for a personalized prevention approach. *J Abnorm Child Psychol* 2018; **46**: 1521–33.

63 Koning NR, Büchner FL, Vermeiren RRJM, Crone MR, Numans ME. Identification of children at risk for mental health problems in primary care—Development of a prediction model with routine health care data. *EClinicalMedicine* 2019; **15**: 89–97.

64 Lewis SJ, Arseneault L, Caspi A, *et al.* The epidemiology of trauma and post-traumatic stress disorder in a representative cohort of young people in England and Wales. *Lancet Psychiatry* 2019; **6**: 247–56.

65 Malda A, Boonstra N, Barf H, *et al.* Individualized prediction of transition to psychosis in 1,676 individuals at clinical high risk: development and validation of a multivariable prediction model based on individual patient data meta-analysis. *Front Psychiatry* 2019; **10**. DOI:10.3389/fpsyt.2019.00345.

66 Moore TM, Calkins ME, Rosen AFG, *et al.* Development of a probability calculator for psychosis risk in children, adolescents, and young adults. *Psychol Med* 2021; : 1–9.

67 Meehan AJ, Latham RM, Arseneault L, Stahl D, Fisher HL, Danese A. Developing an individualized risk calculator for psychopathology among young people victimized during childhood: A population-representative cohort study. *J Affect Disord* 2020; **262**: 90–8.

68 Nichols L, Ryan R, Connor C, Birchwood M, Marshall T. Derivation of a prediction model for a diagnosis of depression in young adults: a matched case-control study using electronic primary care records. *Early Interv Psychiatry* 2018; **12**: 444–55.

69 Pijl MKJ, Bussu G, Charman T, *et al.* Temperament as an early risk marker for autism spectrum disorders? A longitudinal study of high-risk and low-risk infants. *J Autism Dev Disord* 2019; **49**: 1825–36.

70 Rabelo‐da‐Ponte FD, Feiten JG, Mwangi B, *et al.* Early identification of bipolar disorder among young adults – a 22‐year community birth cohort. *Acta Psychiatr Scand* 2020; **142**: 476–85.

71 Rahman R, Kodesh A, Levine SZ, Sandin S, Reichenberg A, Schlessinger A. Identification of newborns at risk for autism using electronic medical records and machine learning. *Eur Psychiatry* 2020; **63**: e22.

72 Rocha TB-M, Fisher HL, Caye A, *et al.* Identifying Adolescents at Risk for Depression: A Prediction Score Performance in Cohorts Based in 3 Different Continents. *J Am Acad Child Adolesc Psychiatry* 2021; **60**: 262–73.

73 Sacrey L-AR, Bryson S, Zwaigenbaum L, *et al.* The Autism Parent Screen for Infants: Predicting risk of autism spectrum disorder based on parent-reported behavior observed at 6–24 months of age. *Autism* 2018; **22**: 322–34.

74 Tate AE, McCabe RC, Larsson H, Lundström S, Lichtenstein P, Kuja-Halkola R. Predicting mental health problems in adolescence using machine learning techniques. *PLOS ONE* 2020; **15**: e0230389.

75 Van Meter AR, Hafeman DM, Merranko J, *et al.* Generalizing the prediction of bipolar disorder onset across high-risk populations. *J Am Acad Child Adolesc Psychiatry* 2020; : S089085672031978X.

76 Elgin DJ. Utilizing predictive modeling to enhance policy and practice through improved identification of at-risk clients: Predicting permanency for foster children. *Child Youth Serv Rev* 2018; **91**: 156–67.

77 Hansmann BC, Eher R. Assisting decisions in child protection service institutions with the RIC – The Risk Indication in Child sexual abuse. *Child Abuse Negl* 2020; **109**: 104652.

78 Thurston H, Miyamoto S. The use of model based recursive partitioning as an analytic tool in child welfare. *Child Abuse Negl* 2018; **79**: 293–301.

79 Vaithianathan R, Rouland B, Putnam-Hornstein E, R. V, B. R. Injury and mortality among children identified as at high risk of maltreatment. *Pediatrics* 2018; **141**: e20172882.

80 Walsh MC, Joyce S, Maloney T, Vaithianathan R. Exploring the protective factors of children and families identified at highest risk of adverse childhood experiences by a predictive risk model: An analysis of the growing up in New Zealand cohort. *Child Youth Serv Rev* 2020; **108**: 104556.

81 Hill RM, Oosterhoff B, Do C. Using machine learning to identify suicide risk: a classification tree approach to prospectively identify adolescent suicide attempters. *Arch Suicide Res* 2019; **10**: 1–18.

82 King CA, Grupp‐Phelan J, Brent D, *et al.* Predicting 3‐month risk for adolescent suicide attempts among pediatric emergency department patients. *J Child Psychol Psychiatry* 2019; **60**: 1055–64.

83 Pettit JW, Buitron V, Green KL. Assessment and management of suicide risk in children and adolescents. *Cogn Behav Pract* 2018; **25**: 460–72.

84 Simon G, Johnson E, Lawrence J, *et al.* Predicting suicide attempts and suicide deaths following outpatient visits using electronic health records. *Am J Psychiatry* 2018; **175**: 951–60.

85 Su C, Aseltine R, Doshi R, Chen K, Rogers SC, Wang F. Machine learning for suicide risk prediction in children and adolescents with electronic health records. *Transl Psychiatry* 2020; **10**: 413.

86 Walsh CG, Ribeiro JD, Franklin JC. Predicting suicide attempts in adolescents with longitudinal clinical data and machine learning. *J Child Psychol Psychiatry* 2018; **59**: 1261–70.

87 Birmaher B, Merranko JA, Gill MK, *et al.* Predicting personalized risk of mood recurrences in youths and young adults with bipolar spectrum disorder. *J Am Acad Child Adolesc Psychiatry* 2020; **59**: 1156–64.

88 Goodwin MS, Mazefsky CA, Ioannidis S, Erdogmus D, Siegel M. Predicting aggression to others in youth with autism using a wearable biosensor. *Autism Res* 2019; **12**: 1286–96.

89 Latham RM, Meehan AJ, Arseneault L, Stahl D, Danese A, Fisher HL. Development of an individualized risk calculator for poor functioning in young people victimized during childhood: A longitudinal cohort study. *Child Abuse Negl* 2019; **98**: 104188.

90 Li H, Jin X, Fan S, *et al.* Behavioural disorders in children with pectus excavatum in China: a retrospective cohort study with propensity score matching and risk prediction model. *Eur J Cardiothorac Surg* 2019; **56**: 596–603.

91 Shui AM, Katz T, Malow BA, Mazurek MO. Predicting sleep problems in children with autism spectrum disorders. *Res Dev Disabil* 2018; **83**: 270–9.

92 van Minde MRC, Blanchette LMG, Raat H, Steegers EAP, de Kroon MLA. Reducing growth and developmental problems in children: Development of an innovative postnatal risk assessment. *PLOS ONE* 2019; **14**: e0217261.

93 Afzali MH, Sunderland M, Stewart S, *et al.* Machine‐learning prediction of adolescent alcohol use: a cross‐study, cross‐cultural validation. *Addiction* 2019; **114**: 662–71.

94 Sylvestre M-P, Hanusaik N, Berger D, *et al.* A tool to identify adolescents at risk of cigarette smoking initiation. *Pediatrics* 2018; **142**: e20173701.

95 Zhang‐James Y, Chen Q, Kuja‐Halkola R, Lichtenstein P, Larsson H, Faraone SV. Machine‐Learning prediction of comorbid substance use disorders in ADHD youth using Swedish registry data. *J Child Psychol Psychiatry* 2020; **61**: 1370–9.

96 Evers O, Schröder P. One size fits all? Die eignung von risikoscreenings zur prognose der inanspruchnahme von angeboten der frühen hilfen. *Prax Kinderpsychol Kinderpsychiatr* 2018; **67**: 462–80.

97 Grebla R, Setyawan J, Park C, *et al.* Examining the heterogeneity of treatment patterns in attention deficit hyperactivity disorder among children and adolescents in the Texas Medicaid population: modeling suboptimal treatment response. *J Med Econ* 2019; **22**: 788–97.

98 Ogrim G, Kropotov JD. Predicting clinical gains and side effects of stimulant medication in pediatric attention-deficit/hyperactivity disorder by combining measures from qEEG and ERPs in a cued GO/NOGO Task. *Clin EEG Neurosci* 2019; **50**: 34–43.

99 Ramsbottom H, Farmer LC. Reducing pediatric psychiatric hospital readmissions and improving quality care through an innovative Readmission Risk Predictor Tool. *J Child Adolesc Psychiatr Nurs* 2018; **31**: 14–22.

100 Yeguez CE, Page TF, Rey Y, Silverman WK, Pettit JW. A cost analysis of a stepped care treatment approach for anxiety disorders in youth. *J Clin Child Adolesc Psychol* 2020; **49**: 549–55.
